# Supplementary figures and images for: In vivo Dicer-2 interactome during viral infection reveals novel pro and antiviral factors in Drosophila melanogaster
Source: PLoS Pathog. 2025 May 7;21(5):e1013093. doi: 10.1371/journal.ppat.1013093 (PMC12058146; doi:10.1371/journal.ppat.1013093)

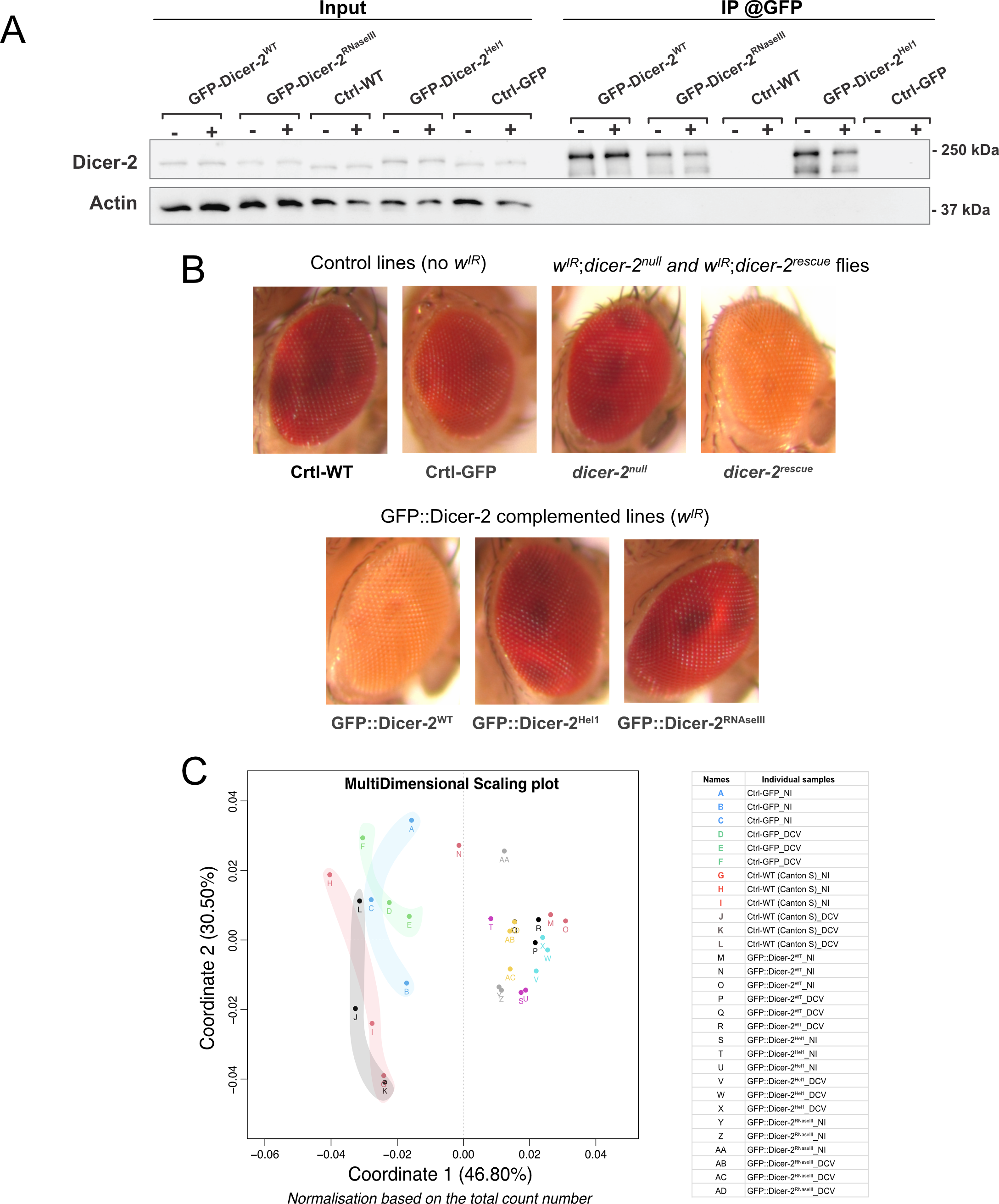

Supplement: S1 Fig — (A) Immunoblot showing the presence of the Dicer-2 in all the samples before immunoprecipitation (IP) using anti-GFP beads, and in the GFP::Dicer-2 samples after IP. The band for the GFP::Dicer-2 fusion protein is slightly higher than Dicer-2 due to the added size molecular weight of the GFP, as expected. Actin, which does not interact with Dicer-2, can be seen in the input but not in the elution. (B) The color of the eyes of wIR flies allow the monitoring of RNAi efficiency. The dicer-2null and dicer-2rescue flies are described in Kemp et al., 2013 [6]. Flies without the transgene normally have red eyes (see Ctrl-WT and Ctrl-GFP). When the flies contain the wIR transgene, if RNAi is efficient, it induces KD of the white gene and results in a white eye phenotype (see dicer-2rescue) and if RNAi is inefficient the KD is not effective and eyes are red like WT flies (see dicer-2null). RNAi works normally in GFP::Dicer-2WT, but not GFP::Dicer-2Hel1 or GFP::Dicer-2RNaseIII flies. Photos, by C. Meignin, are under CC BY 4.0 license. (C) Multidimensional Scaling (MDS) analysis showing the five genotypes in non-infected (NI) and infected (DCV) conditions tested in triplicate. This plot visualizes the overall distances between samples with normalization based on the total count number. Control lines (Crtl-WT and Crtl-GFP) cluster together but distinctly from the Dicer-2::GFP samples, reflecting their unique characteristics. Non-infected and DCV infection show no clear separation. See S1 Text. (TIFF) [file ppat.1013093.s001.tiff]

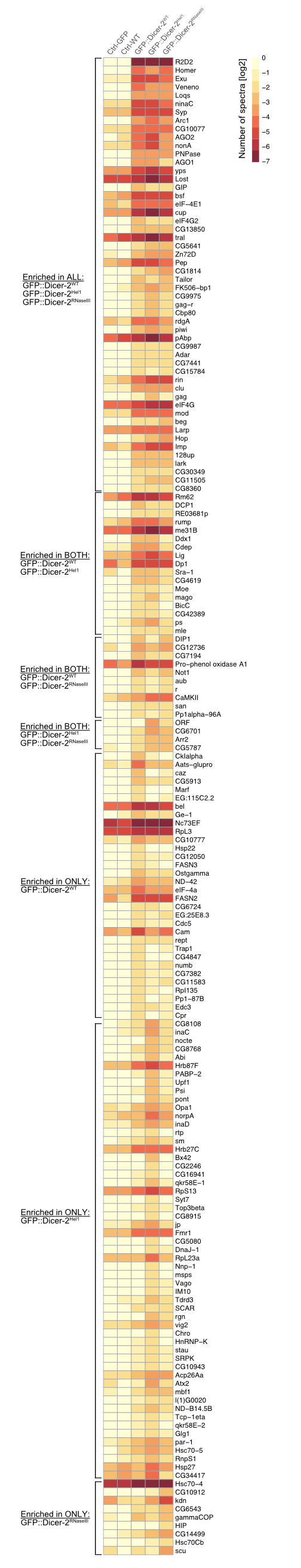

Supplement: S2 Fig — Heatmap representing the number of spectra of the proteins enriched in any of the three GFP::Dicer-2 lines (Fig 1C-E), separated by the categories of the Venn diagram in Fig 1F. (TIFF) [file ppat.1013093.s002.tiff]

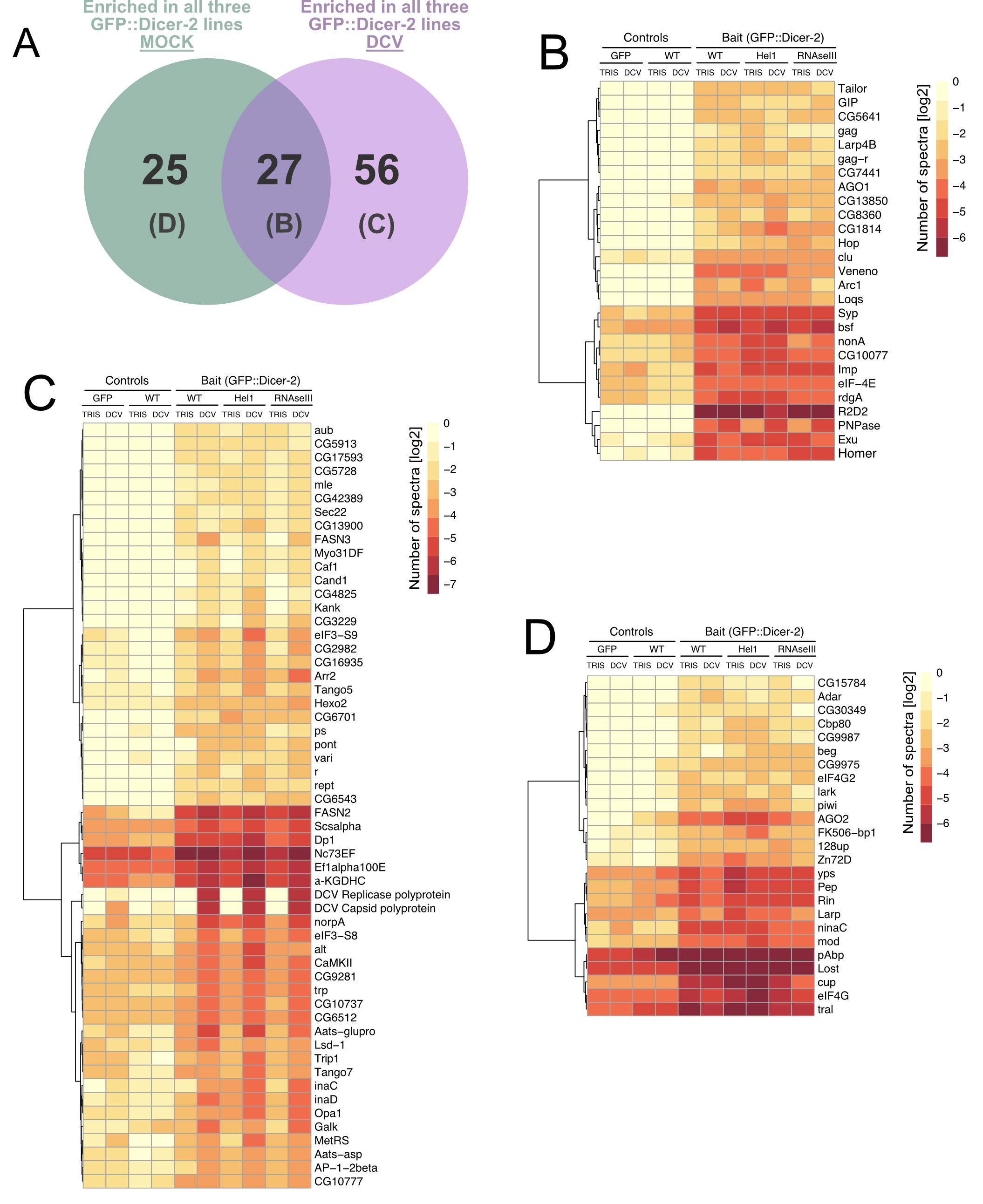

Supplement: S3 Fig — (A) Venn diagram showing the number of proteins identified in all three GFP::Dicer-2 lines in mock-condition (Fig 1F, 52 proteins) and/or all proteins identified in all GFP::Dicer-2 lines in DCV-infected adult flies (Fig 2D, 83 proteins). (B-D) Heatmaps representing the number of spectra of the proteins enriched in the different categories depicted in (A). Clustering was performed using the Ward method. (TIFF) [file ppat.1013093.s003.tiff]

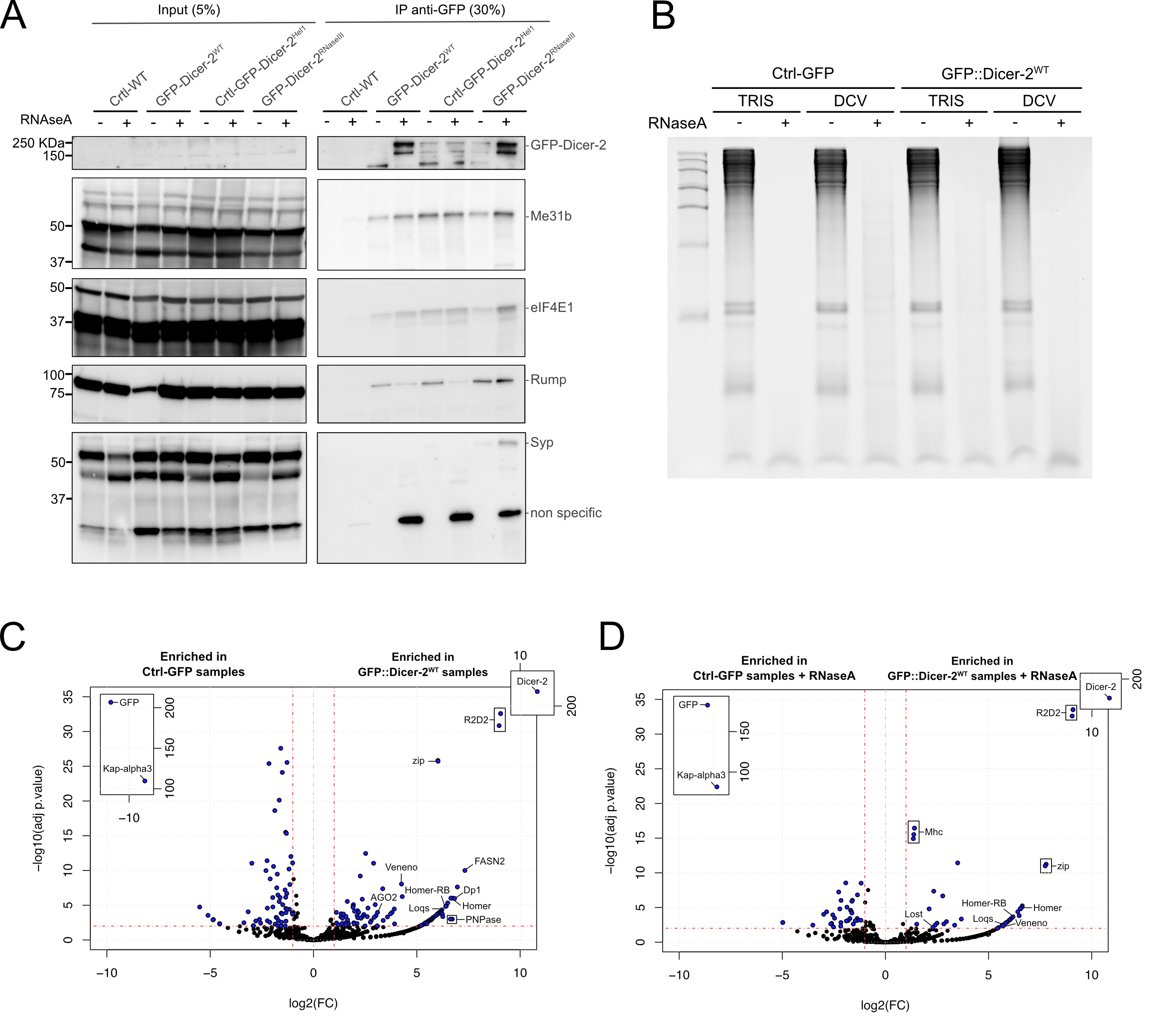

Supplement: S4 Fig — (A) Immunoprecipitation of (A): GFP::Dicer-2WT or, GFP::Dicer-2Hel1 or GFP::Dicer-2RNAseIII, in comparison to wild-type CantonS flies (Ctrl-WT) with (+) or without (-) RNase A treatment. Representative of three independent experiments (n = 3) except for Dicer-2RNaseIII (n = 1). (B) Urea/Acrylamide gel showing total RNA after no treatment (-) or treatment with 15 µg of RNase A (+). Adult flies were injected or not with DCV and protein extraction was performed in the same manner as for the IPs. Instead of proceeding with the IP, total RNA was extracted after no treatment or treatment with RNase A to confirm the efficiency of the RNase treatment. (C, D) Volcano plots representing the fold changes and adjusted p-value of the GFP::Dicer-2WT partners versus the control line (Ctrl-GFP) either without (C) or with (D) RNase A treatment. Fold-changes and adjusted p-values (adjp) were obtained using a negative binomial test and p-values were corrected by the Benjamini-Hochberg method to obtain adjusted p-values. All the proteins with fold change > 2 and an adjusted p-value < 0.01 are represented in blue. (TIFF) [file ppat.1013093.s004.tiff]

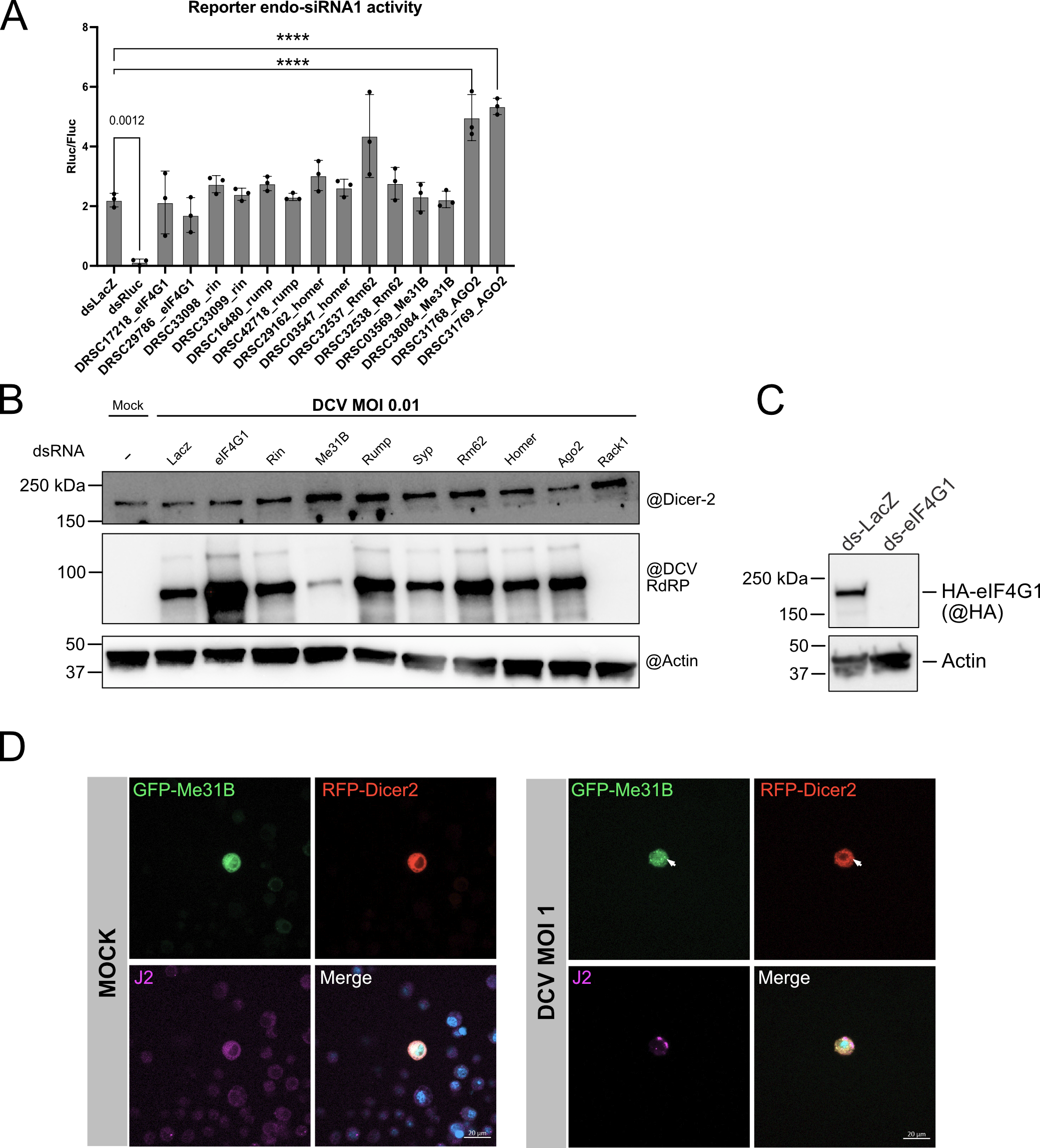

Supplement: S5 Fig — (A) Candidates depleted S2 cells were transfected with endo-siRNA1 Renilla luciferase reporters together with firefly luciferase (transfection control). Renilla luciferase counts (Rluc) were normalized using firefly luciferase counts (Fluc) (n = 3 independent experiments). Mean ± standard deviation (SD). Statistical significance was analysed using one-way ANOVA followed by Dunnett’s multiple comparison test. “****” for p < 0.0001. (B) Immunoblot of DCV RdRp in S2 cells after knockdown of Dicer-2’s partners. DCV RdRp is only detected in infected cells excepted dsRNA RACK1 treated cells which served as positive control. Representative of two independent experiments. (C) S2 cells treated with dsRNA targeting eIF4G1 inhibits the expression of HA-eIF4G1. Representative of two independent experiments. Actin antibody is used as loading control. (D) Co-expression of RFP-Dicer-2 and GFP-Me31B reveals co-localisation in S2 cells during DCV infection. Arrows indicate cytoplasmic granules with the two proteins. dsRNA are stained with J2 antibody. Transfected cells were infected at DCV MOI1 during 24h. blue = DAPI. Representative of two independent experiments. scale = 20µm. (TIFF) [file ppat.1013093.s005.tiff]

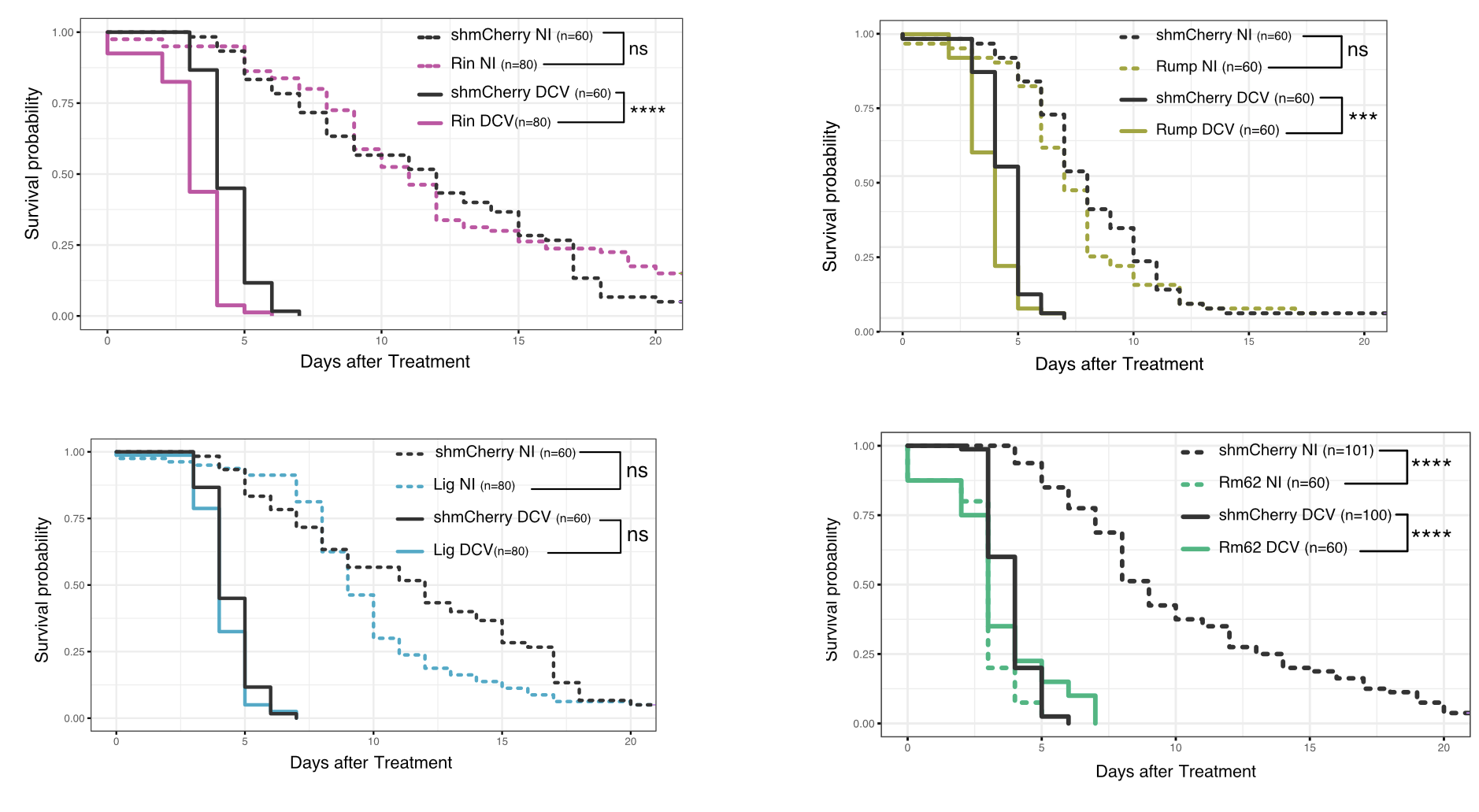

Supplement: S6 Fig — Survival analysis of Rin, Rump, Lig and Rm62 knockdowns in vivo after infection with DCV 50 pfu. Data were collected from three or four independent experiments, each comprising three groups of 20 flies (n = number of individual flies is indicated). Log-rank test with Benjamini-Hochberg (BH) correction was used for multiple comparisons of survival curves in each batch. Non infected (NI) and DCV infected conditions for each survival were compared to shmCherry line. The p-values are represented as follows: “***” for adj.p-val < 0.001, “****” for adj.p-val < 0.0001 and “ns” for non significant. (TIFF) [file ppat.1013093.s006.tiff]

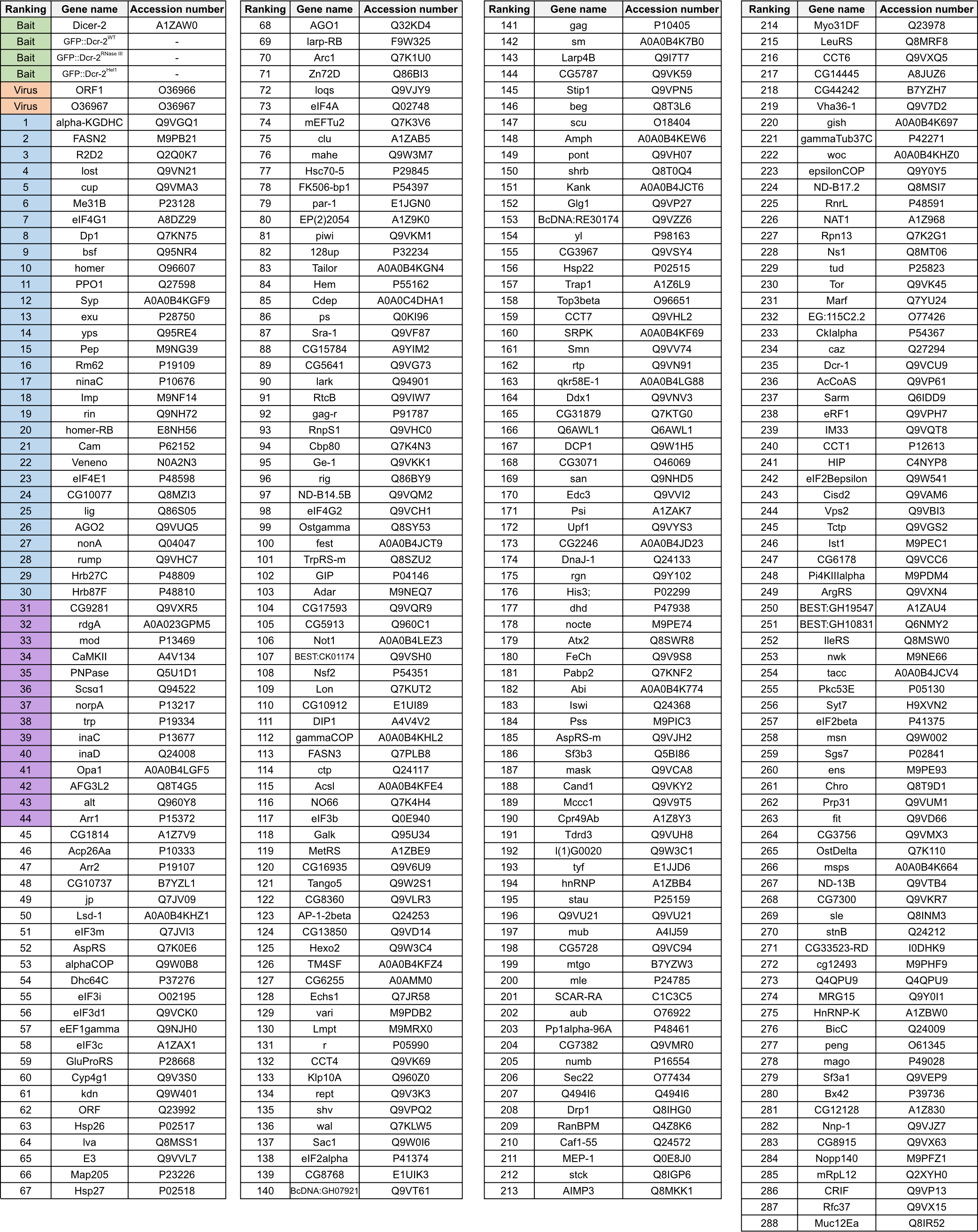

Supplement: S2 Table — List of 288 proteins that were highlighted using the SAINTexpress tool. The baits are represented in green, two viral proteins are in orange, the top 10% candidates are in blue, and the top 15% are in purple. (TIFF) [file ppat.1013093.s008.tiff]

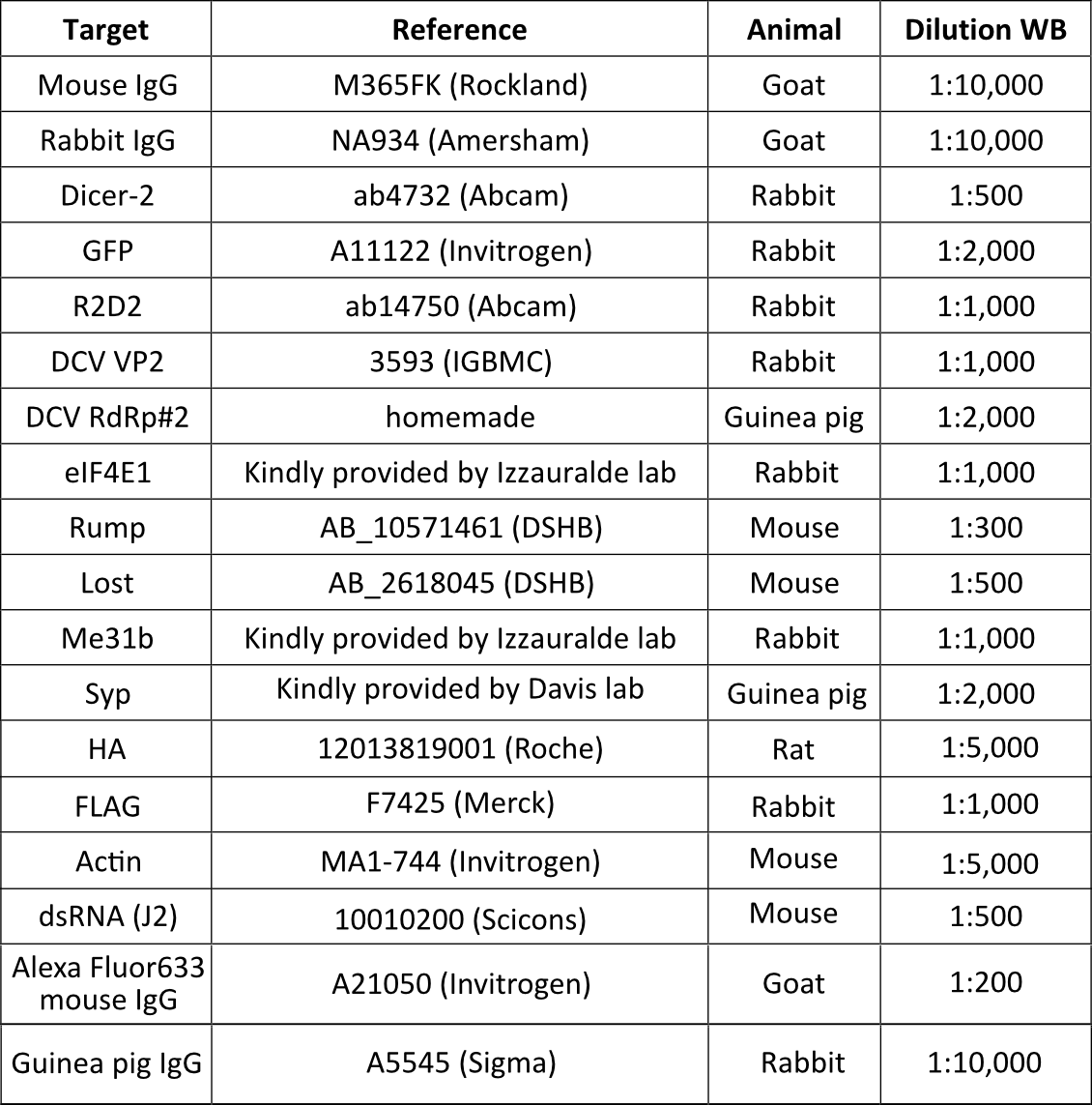

Supplement: S3 Table — (TIFF) [file ppat.1013093.s009.tiff]

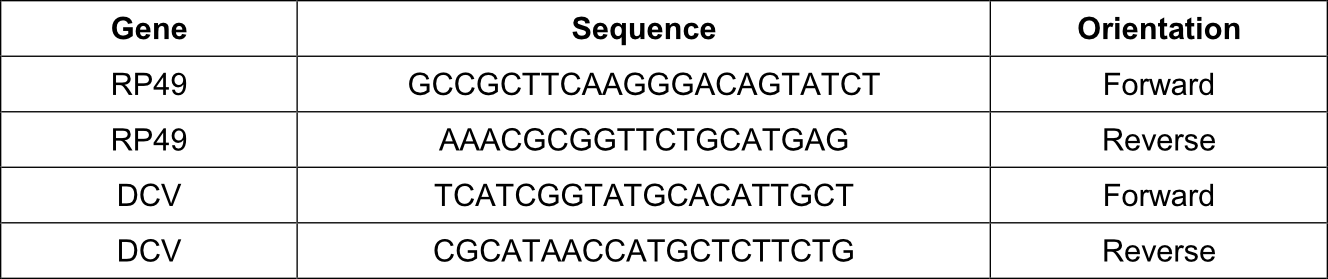

Supplement: S4 Table — (TIFF) [file ppat.1013093.s010.tiff]

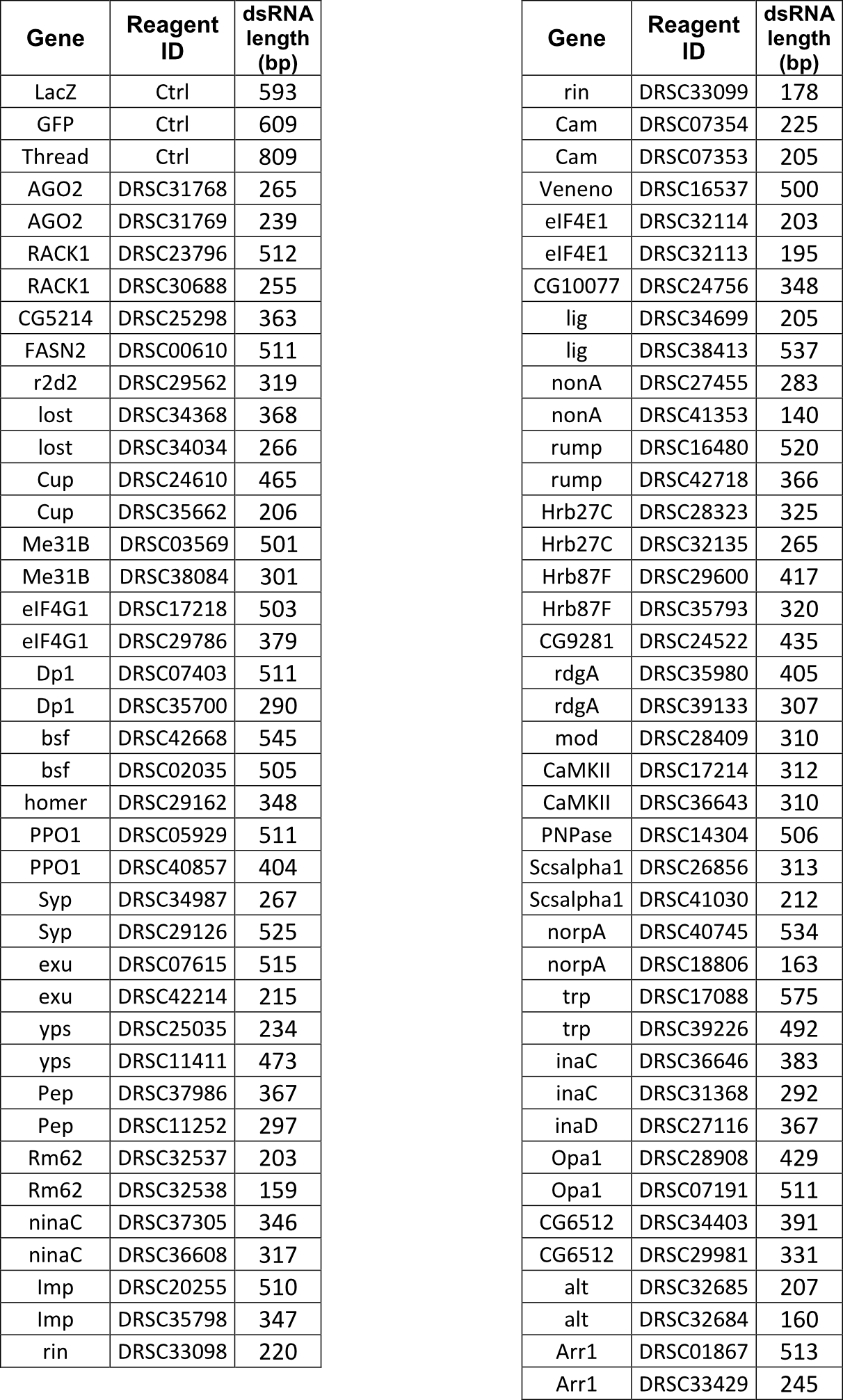

Supplement: S5 Table — (TIFF) [file ppat.1013093.s011.tiff]
