# Supplementary material for: In vivo Dicer-2 interactome during viral infection reveals novel pro and antiviral factors in Drosophila melanogaster
Source: PLoS Pathog. 2025 May 7;21(5):e1013093. doi: 10.1371/journal.ppat.1013093 (PMC12058146; doi:10.1371/journal.ppat.1013093)
Supplement: S1 Text — (PDF) [file ppat.1013093.s012.pdf]

Supplemental procedures

Plasmids and cloning methods

The plasmids encoding GFP-Me31B (#21682), GFP-Rin (#146349), HA-eIF4G1 (#79259) under the Ac5 promoter were provided by Addgene and their reference number are indicated in brackets. The plasmid coding for HA-eIF4G1 (#79259) contained a frame-shifting mutation which was corrected by site-directed mutagenesis. The punctual mutations in GFP-Rin were similarly introduced. The vectors expressing Flag-Dicer-2, GFP-Dicer-2 and RFP-Dicer-2 were already described (Girardi *et al*, 2015).

The luciferase reporters for endosiRNA1 and endosiRNA2 were designed based on the target sequences for esi1_2 and esi2_1, as described by Czech and colleagues (Czech *et al*, 2008). These sequences were specifically reconstructed by annealing primers containing the corresponding target sequences along with restriction sites, followed by their insertion into a pAC5.1-RLuc-V5His6 (pAc-*Renilla*) vector that had been previously linearized using the restriction enzymes XhoI and BamHI. pAC5.1-RLuc-V5His6 and pAC5.1-FLuc-V5His6 was a gift from Elisa Izaurralde (Addgene plasmids #21182 and #21183).

Cell Culture, Transfection, infection

S2 cells are transfected with expression plasmids by lipofection using the Effectene Transfection Reagent Kit (Qiagen), according to the manufacturer's instructions.

For immunolocalisation, S2 cells (5×10^6 cells/well) were seeded in a 24-well plate with 400 µL of complete medium, and transfected with 200 ng of plasmid encoding either EGFP-Me31B, EGFP-Rin, EGFP-Rin^{S142E}, or EGFP-Rin^{S142A}, 200 ng of mRFP Dicer-2, and 100 ng of empty pAc5.1 plasmid. Transfected cells were incubated at 25°C for 48 hours, then infected with Drosophila C virus (DCV) at a multiplicity of infection (MOI) of 1. After a 1-hour adsorption with regular agitation, the viral medium was replaced with 500 µL of complete Schneider's medium, and cells were incubated for an additional 24 hours.

To assess the knockdown efficacy, depleted-S2 cells were counted and seeded into a 24-well plate 3 days after dsRNA treatment. The cells were then transfected with 150 ng of HA-eIF4G1 expression plasmid. Samples were collected 48 hours post-transfection to measure the protein expression levels by western blot.

For the luciferase assays, 3 days after dsRNA treatment, S2 cells are re-seeded in 24-well plate, and thus transfected with a mixture containing 75 ng of endosiRNA1 or endosiRNA2 *Renilla* luciferase (Rluc) reporter, 25 ng of firefly luciferase (Fluc) plasmid as transfection control and 100 ng of the corresponding dsRNA.

For co-immunoprecipitation, S2 cells were seeded in 6-well plates (3.10^6 cells/well, 2 wells/conditions) and transiently transfected with a mixture containing plasmids expressing GFP-tagged and HA-tagged or FLAG-tagged protein (0.25-1 µg/well/plasmid).
To monitor DCV infection, S2 cells were infected after 3 days of dsRNA treatment with DCV (MOI = 0.01) in 250 µL of viral medium for 1 hour with agitation. The viral medium was then removed, and replaced with 500 µL complete Schneider's medium for an additional 24 hours. Cells were harvested 24 hours post-infection for western blot analysis.

dsRNA Production

dsRNA templates were obtained from the Drosophila RNAi Screening Center (DRSC) and amplified by PCR using T7 primers (5' TAATACGACTCACTATAG 3') and Phusion High-Fidelity DNA Polymerase (Thermo Fisher Scientific). The PCR products were purified using the Qiagen Gel Purification Kit and used as templates for in vitro transcription (IVT) with the MEGAscript T7 Kit (Thermo Fisher Scientific), following the manufacturer's protocol. After transcription, RNA was treated with DNase and purified using phenol-chloroform extraction. The dsRNA was annealed by heating at 95°C for 5 minutes, followed by slow cooling to room temperature.

RNA Interference in S2 cells

S2 cells (5×10^5 cells/well) were seeded in 24-well plates with 250 µL starvation medium (Schneider's Drosophila medium supplemented with 1% penicillin/streptomycin and 1% Glutamax) and treated with 6 µg of dsRNA for 3 hours. The medium was then complemented with Schneider's medium containing 20% FBS, 1% penicillin/streptomycin, and 1% Glutamax. After 72 hours, cells were infected with DCV (MOI = 0.01) in 250 µL of viral medium for 1 hour with agitation. The viral medium was removed, and cells were incubated with 500 µL complete Schneider's medium for an additional 24 hours. Cells were harvested 24 hours post-infection for western blot analysis.

Protein Extraction and SDS-PAGE

Cells were harvested 24 hours post-infection, washed with PBS, and resuspended in 2x protein sample buffer (100 mM Tris HCl [pH 6.8], 4% SDS, 20% glycerol, 0.2 M DTT, 0.5% bromophenol blue) or 2X Laemmli SDS buffer (Alfa Aesar) supplemented with 0.1 M DTT. Samples were boiled at 95°C for 5 minutes, centrifuged, and loaded onto Mini-PROTEAN TGX Precast Gels (Bio-Rad) for SDS-PAGE. Protein electrophoresis and subsequent steps were performed as previously described.

Co-Immunoprecipitation in S2 cells

Two or three days after transfection, cells were harvested, washed once with ice-cold phosphate-buffered saline (1× PBS), and resuspended in 0.5 ml of NET buffer (50 mM Tris HCl [pH 7.4], 50 mM NaCl, 1 mM EDTA, 0.1% NP40), containing 5% glycerol and supplemented with Complete-EDTA-free Protease Inhibitor Cocktail (Roche). Cells were lysed by three 30s sonication (Bioruptor Plus, Diagenode) and spun for 15 min at 16 000 g and 4°C to pellet cells debris. After setting aside 50 µl of the cleared lysate to serve as input, the supernatant was completed with 500 µl of NET buffer and with 20 µl of GFP-Trap Agarose beads (ChromoTek), and samples were then rotated for 2-4 hours (12 rpm) on a wheel at 4°C. Then, samples beads were washed three times with 0.5 mL of NET buffer, followed by a fourth wash in NP40-free NET buffer. Bound proteins were finally eluted with 75 µl of 2x protein sample buffer and analyzed by western blot.

Dual-Luciferase Assay

Dual-luciferase assays were performed using the Promega Dual-Luciferase Reporter Assay System following the manufacturer's instructions. At 48 hours post-transfection, cells were harvested, and luminescence was measured using a Varioskan Lux microplate reader (10,000 ms reading time for both firefly and *Renilla* luciferase activities). Data were analyzed using GraphPad Prism 10.

Immunofluorescence Staining

At 24 hours post-infection, 3×10^5 cells were seeded into Lab-Tek II 8-chamber slides (Thermo Scientific) pretreated with concanavalin A (10 mg/mL in PBS) for 1 hour. Cells were fixed with 4% paraformaldehyde (Electron Microscopy Sciences) for 15 minutes, permeabilized with PBS containing 0.1% Triton X-100 (PBTx) for 10 minutes, and blocked with PBTx containing 1% BSA for 1 hour. After blocking, cells were incubated with primary antibody (anti-dsRNA J2, 1/500) overnight at 4°C. Cells were washed twice with PBTx and then incubated with the secondary antibody (goat anti-mouse Alexa Fluor 633, 1/200) for 1 hour at room temperature in a dark container. After further washing, 30 µL of Vectashield Antifade mounting medium (Vector Laboratories) with NucBlue Live ready probe (Invitrogen) (1:1, v/v) was added per well, and slides were sealed with coverslips.

Imaging

Eye images were taken on a Zeiss Stereo Discovery V12. Confocal images were acquired using a Zeiss LSM980 confocal microscope with a 60× oil immersion objective. Fluorescence excitation was achieved using lasers at 353 nm (DAPI), 488 nm (EGFP), 590 nm (mRFP), and 633 nm (Alexa Fluor 633), with image processing using ZEN Blue software (Zeiss).

Cloning primers (5’-3’)

HA-eIF4G1 correction:

F: CAAAGCACCGCAAAGGAAAAAAAATTACAGCCAATTGTGGATAAAATTAAG

R: CTTAATTTTATCCACAATTGGCTGTAATTTTTTTTCCTTTGCGGTGCTTTG

Rin-S142A mutagenesis:

F: GATGGCGAGGCGCGATCGGAG ; R: CTCCGATCGCGCCTCGCCATC

Rin-S142E mutagenesis:

F: GATGGCGAGGAGCGATCGGAG ; R: CTCCGATCGCTCCTCGCCATC

EndosiRNA1 reporter:

F: TCGAGcaacagtttatttacttggaggcaacataatcaaatgaactgagggttacttggagg caacataatcaG

R :GATCCtgattatgttgcctccaagtaaccctcagttcatttgattatgttgcctccaagtaaa taaactgttgC

EndosiRNA2 reporter:

F:TCGAGcaacagtttattggagcgaacttgttggagtcaaaatgaactgagggtggagcgaa cttgttggagtcaaG

R: GATCCttgactccaacaagttcgctccaccctcagttcattttgactccaacaagttcgctcc aataaactgttgC

Acknowledgments:

Figure 1B includes images sourced from smart.servier.com (https://smart.servier.com/smart_image/smart-microtube/), licensed under CC BY 4.0. Other icons were created using Affinity Designer V1.

Czech B, Malone CD, Zhou R, Stark A, Schlingeheyde C, Dus M, Perrimon N, Kellis M, Wohlschlegel JA, Sachidanandam R, *et al* (2008) An endogenous small interfering RNA pathway in Drosophila. *Nature* 453: 798–802

Girardi E, Lefèvre M, Chane-Woon-Ming B, Paro S, Claydon B, Imler J-L, Meignin C & Pfeffer S (2015) Cross-species comparative analysis of Dicer proteins during Sindbis virus infection. *Sci Rep* 5: 10693
