# Supplementary material for: In vivo Dicer-2 interactome during viral infection reveals novel pro and antiviral factors in Drosophila melanogaster
Source: PLoS Pathog. 2025 May 7;21(5):e1013093. doi: 10.1371/journal.ppat.1013093 (PMC12058146; doi:10.1371/journal.ppat.1013093)
Supplement: Attachment — Submitted filename: 2_2024_reviewComments.pdf [file ppat.1013093.s014.pdf]

submission PPATHOGENS-D-24-00621

In vivo Dicer-2 interactome during viral infection reveals novel pro and antiviral factors in *Drosophila melanogaster*

By Rousseau et al.

We thank the reviewers for their constructive and insightful comments, which have been instrumental in guiding us to improve the manuscript in meaningful ways. In general, we appreciate the recognition of the value our study brings to the field of insect immunity and the acknowledgment of the innovative aspects of our approach, including the profiling the Dicer-2 interactome *in vivo* and under infection conditions. We have taken into account the remark that additional data analysis and experiments are necessary to strengthen our conclusions, and have revised the manuscript accordingly. In particular, we have restructured the entire MS analysis to follow the suggestion of Reviewer#1 to analyze the non-infected and infected samples independently and in more detail, thereby strengthening our analysis. Moreover, we have performed some additional experiments focusing on Dicer-2's partners. We believe these revisions address the issues raised by the reviewers and significantly improve the clarity, robustness and impact of the manuscript.

Point-by point response to the Reviewers' comments: All modifications in the manuscript are highlighted in red

Reviewers' comments

Part II – Major Issues: Key Experiments Required for Acceptance

Reviewer #1:

General reply: We would first like to thank Reviewer#1 for their comments. We appreciate in particular the recognition of the merits of our approach. In addition, we are thankful for the suggestions made on how to improve the manuscript.

I was puzzled by the choice of negative controls. Why has no ubi>GFP line been used that matches the genetic background of the rescue lines? Is the Canton S fly of the same genetic background as the transgene expressing lines? This was not clear to me and should be better explained in the methods section. If the negative control lines are of a different background, this is not ideal for the analysis. In this case, can the authors comment on the question whether protein expression of distinct genetic backgrounds is comparable, perhaps using published proteomics data, if available.

Reply: *We used CantonS flies as a control line because all transgenic lines used in the manuscript contained a white inverted repeat (w^{IR}) transgene on the X-chromosome in a CantonS genetic background. The w^{IR} transgene allows us to follow RNAi deficient genotype using the eye color phenotype as illustrated in Fig. S1A. The w^{IR} transgene, inserted in CantonS X-chromosome, was*

described in Lee et al. 1998 where dicer-2 alleles (dicer-2^{L811fsX} used in this manuscript) were described (PMID: 15066283). These flies are not isogenized in a specific genetic background, and there would therefore be no perfect control for them, which is why we have decided to use the CantonS flies as the next best alternative. Finally, we have previously shown that mutant dicer-2 complemented by GFP::Dicer-2^{WT} phenocopy CantonS flies when it comes to RNAi (PMID: 26024431). These precisions are implemented in the Materiel and Methods section p.20, line 516.

To get a reliable dataset and avoiding non-specific interaction with GFP protein, we decided to include transgenic flies expressing GFP alone using transgenic line P{UAS-GFP.S65T}Myo31DF[T2] line (BDSC #1521) crossed with actin5C promoter controlling Gal4 driver (BDSC #25374).

Additionally, we performed a Multidimensional Scaling (MDS) analysis to visualize the similarity and dissimilarity between mass spectrometry samples in a reduced-dimensional space (Figure below). Control lines (CantonS "CTRL_DICER_CS" and GFP-expressing flies "CTRL_GFP") cluster together but distinctly from the Dicer-2::GFP samples (J to AD, Figure below), reflecting their unique characteristics. We used CantonS and GFP-expressing flies as control lines for mass spectrometry analysis.

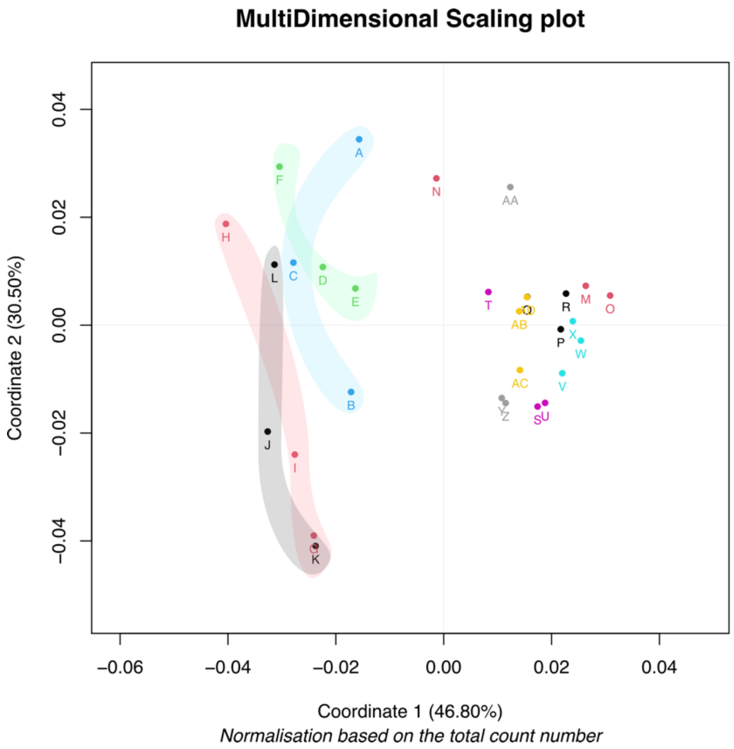

Libnames	group
A	CTRL_GFP_NI
B	CTRL_GFP_NI
C	CTRL_GFP_NI
D	CTRL_GFP_DCV
E	CTRL_GFP_DCV
F	CTRL_GFP_DCV
G	CTRL_DICER_CS_NI
H	CTRL_DICER_CS_NI
I	CTRL_DICER_CS_NI
J	CTRL_DICER_CS_DCV
K	CTRL_DICER_CS_DCV
L	CTRL_DICER_CS_DCV
M	DICER_WT_NI
N	DICER_WT_NI
O	DICER_WT_NI
P	DICER_WT_DCV
Q	DICER_WT_DCV
R	DICER_WT_DCV
S	DICER_Hel1_NI
T	DICER_Hel1_NI
U	DICER_Hel1_NI
V	DICER_Hel1_DCV
W	DICER_Hel1_DCV
X	DICER_Hel1_DCV
Y	DICER_RNaseIII_NI
Z	DICER_RNaseIII_NI
AA	DICER_RNaseIII_NI
AB	DICER_RNaseIII_DCV
AC	DICER_RNaseIII_DCV
AD	DICER_RNaseIII_DCV

Figure to the reviewer comments: Multidimensional Scaling (MDS) Analysis of Mass Spectrometry Samples.

The description of mass spec data analysis is hard to follow and should be clarified. It is unclear to me, how the authors distinguish the "overall/global" Dicer-2 analysis from the specific analysis of the wild-type construct and the two mutant constructs. Also in the global analysis shown in Fig 1C and S1C, it is possible to distinguish differential enrichment of interacting partners to different constructs in the mock and virus infected condition. Also the purpose of an overall analysis is not entirely clear to me, given

that the introduction of the manuscript highlights the molecular difference between the distinct Dicer-2 constructs.

The authors present the analysis of overall Dicer interactome and the specific analysis of mutants as "two different methods" (line 209) which I find quite a stretch given that the experimental input for these analyses are the same. Due to this double analysis presented in figures 1 and 2 it becomes very hard to follow which protein factors the authors consider general interaction partners and more specific factors that are interacting in a construct/infection dependent status. Given that the authors consider their data a resource to the community, it would be ideal to have an easy-to-interpret representation of the data. I would recommend to analyze the interactome of wild-type and mutant mass spec data separately in uninfected and infected cells (as done for fig 2, I assume). These data should be shown as a figure for all constructs, not only the wild type as an example. Also a supplemental table with raw mass spec, akin to the table of candidates in Suppl. Table 1 data but then for all interactors in each condition would be helpful for the interested reader, who wants to use the data as resource. When the interaction partners in individual conditions are established, overlapping factors can be identified between mutants and wildtype in the uninfected and infected conditions separately. Only hereafter, I would recommend to dive into the interaction of Dicer-2 networks being modulated by DCV infection. Ideally this should be done using a plot that shows the underlying data (for instance a scatter-plot showing correlation between noninfected and DCV infected samples) Venn diagrams by themselves are not particularly informative. The authors may find a different order of analysis more useful, but it is highly recommended to reorder the line of thoughts, as the current representation blurs main messages. I think this is a missed chance.

Reply: *We would first like to thank Reviewer#1 again for their insightful comment on how to improve and clarify our mass spectrometry analysis. We agree with the suggested line of thoughts, and have therefore restructured the text and figures in order to analyze the WT and mutant data separately in non-infected and infected conditions. The new order of analysis is as follows:*

***(1)** we first focused only on the non-infected (mock) samples and have, for each GFP::Dicer-2 line, compared the proteins enriched in the GFP::Dicer-2 samples to the controls (Ctrl-WT + Ctrl-GFP). This analysis is described in the text in the paragraph titled "Comparative analysis of the interaction profiles of Dicer-2^{WT} and mutants". The data is represented as a volcano plot for each GFP::Dicer-2 line (Figure 1C-E), a Venn diagram (Figure 1F) and a heatmap representing the number of spectra for the proteins of each Venn diagram category. **(2)** We then focused on the infected samples only, and performed the same comparison for each GFP::Dicer-2 line. This analysis is described in the text in the paragraph titled "DCV infection strongly alters the interactome of Dicer-2^{WT} and its mutants". The data is again represented as a volcano plot for each GFP::Dicer-2 line and a Venn diagram (Figure 2). Furthermore, we have added in the new Suppl. Fig. 2 a comparison of the interaction profiles of each fly line in non-infected vs infected conditions focusing on proteins enriched in all three GFP::Dicer-2 lines in mock samples only, mock AND infected samples, or infected samples only. The data is represented as a Venn diagram and three heatmaps representing the number of spectra for the proteins of all categories. **(3)** Finally, we used the SAINT analysis (previously "Global analysis") to have a way to visualize the*

cumulative impact of both the mutations and the infection in one easy-to-understand dot plot, and to establish a list of candidates to validate further, which includes most of the main candidates from the previous analyses. This SAINT analysis is described in the text in the paragraph titled "Visualization of the differential interaction profiles of Dicer-2 and generation of a candidate list". The data is represented as a dot plot and a Cytoscape network (Figure 3).

As Reviewer#1 stated that the purpose of the overall analysis was unclear for them, we would like to clarify that the purpose of this SAINT analysis was to be able to establish a list of candidates to validate further. Indeed, for most of the different categories highlighted by the different line-specific analyses, a rational for validating the candidates could be found. As the main purpose of this work was to provide a resource to the scientific community, the validation present in this manuscript was performed mainly to validate the quality of our interactome, and the potential of the different proteins highlighted in each category to be of relevance to scientists in the field. Therefore, instead of cherry-picking interesting candidates from every category, we decided to use this SAINT analysis to have an unbiased list of candidates to further validate, which contains most of the main candidates highlighted in the different categories. We therefore need to include it in the manuscript, but we hope that the modifications that we made in the text corresponding to this analysis will render the purpose of this analysis clearer. We hope that our line-specific analyses will be of use for our colleagues who may wish to investigate further the other candidates present in the different categories.

In conclusion, we have tried to follow as best as we could the recommendations of Reviewer#1 by analyzing the mass spectrometry data independently for non-infected and infected samples, which allowed us to focus first on the impact of the mutation on the interactome of Dicer-2, and then on the impact of the infection. We also have included the results for each GFP::Dicer-2 line as an individual volcano plot, as suggested, and added a supplementary figure containing the interactants highlighted by all analyses. Finally, we have clarified the purpose of the SAINT analysis. We believe that, by following these suggestions, we were able to strengthen our mass spectrometry data analysis. All raw datasets are provided in Supplementary Table 1. We will make all raw data and scripts available on Figshare with a DOI that will be made available upon publication of the manuscript (temporary private link: https://figshare.com/s/442b2f133f6c16b538cb).

The choice of top 15% to define candidates and predict interaction networks is quite random. Well known interactors of Dicer-2 like Loqs are identified but have a lower rank number, suggesting that even a less stringent analysis can possibly identify relevant Dicer-2 interactors. The argument that 36 genes out of the 44 overall Dicer interactors are shared between the 146 proteins common to all constructs IPs is not an independent proof, as the analyses rely on the same data.

Reply: *As mentioned above, the main purpose of this paper is to provide a panel of candidates that can be further studied by our fellow researchers, depending on their specific interests. Indeed, a justification for the study of almost all of the categories highlighted by our analyses could be found. Thus, we wanted*

a way to test some candidates from several categories, and this is why we decided to perform the SAINT analysis, which is often used when an MS experiment requires several bait proteins. As described in the submitted manuscript, we selected the top 15% of proteins (i.e., the top 44 candidates) following the SAINT analysis to establish a network using the STRING database. However, this should not be considered as an exhaustive list of Dicer-2 interactants, but rather as a subset of potential interactants that we used to validate the robustness of our MS experiment, due to the diversity of their interaction profiles. To avoid confusion between the SAINT analysis and the line-specific analyses in non-infected and DCV-infected conditions (new Figures 1 and 2), we now refer to the "overall/global" Dicer-2 analysis as "selected candidates from the Dicer-2 interactome network" in, the revised manuscript. This also more accurately depicts our intention of using these candidates to validate the interactome. Among the 44 selected candidates, most are shared between the non-infected and/or DCV-infected conditions. However, we agree that, since these analyses were performed using the same set of raw data, comparing the number of proteins that are shared may not be particularly informative. For this reason, we have not emphasized it in our revised manuscript. Finally, the 15% threshold was chosen because it provided a manageable number (44 candidates) for further validation in S2 cells and in vivo experiments. Indeed, this number represents the maximum number of candidates that we had the capacity to further validate in the lab. As performing an in vivo screen in drosophila is quite labor-intensive, we were unfortunately limited in the number of candidates that we could realistically validate.

Regarding the partner Loqs, we were also surprised not to find Loqs among the top-ranked candidates, like R2D2, given that Loqs is a well-known and validated interactant of Dicer-2. However, while Loqs is not in the top 15% of selected candidates, it appears among the 27 common interactants shared across the three GFP::Dicer-2 lines, regardless of conditions (as shown in the new analysis, Supplementary Figure S2B). Endogenous Loqs-PC/PD was first identified as a partner of Dicer-2 by Zou et al. 2009 (PMID: 19635780). It is worth noting that previous immunoprecipitation (IP) experiments have primarily been conducted in S2 cells, using overexpressed Flag-Dicer-2 as bait, where Loqs proteins are well expressed (PMID: 19635780). Two datasets involving Dicer-2 as bait in flies have been reported. Marques et al. 2010 (PMID: 20037596) used a Dicer-2-HA construct (a genomic rescuer of Dicer-2 in a null mutant background) in adult flies and found that Loqs does not co-immunoprecipitate with Dicer-2-HA, even though it is present in the input (Supplementary Figure 2C of paper cited). Similarly, Nadimpalli et al. 2022 (PMID: 36114004) immunoprecipitated endogenous Dicer-2 in embryos, and Loqs was not identified as a "high-confidence interactor" in their dataset (Supplementary Table of paper cited). The low abundance of peptides detected in adult flies, even if consistent, following GFP::Dicer-2 IP may explain Loqs' relatively low ranking (72nd position) in our dataset compared to R2D2, which ranked in 3rd position.

It is odd to calculate an overall enrichment for Dicer interactors upon DCV infection (Fig 2A). Figure S1A shows that an enrichment upon infection can be different for each Dicer-2 construct (e.g. NinaC, Cup, Me31B). By analyzing DCV enrichment as a whole, one loses this important information. As indicated

in my previous comment, the interacting partners should first be identified separately in different conditions to take this analysis as a basis for further comparisons.

Reply: *We believe we have addressed this concern in our revised manuscript, where we analyze non-infected and DCV-infected conditions separately, as presented in the new Figures 1 and 2. These figures allow for a clearer comparison of interacting partners under each condition and avoid potential loss of information due to aggregation.*

The authors should explain their choice for the proteins that are validated by Western blot in Fig 3. Now it feels random. The figure would have more added value, if more of the candidates that are later followed upon had been included (for example: eIF4G, rin, lig)

Reply: *We appreciate the Reviewer's suggestion and understand how the selection of proteins for validation, previously in Figure 3 (new Figure 4), may appear random. However, the availability of antibodies for drosophila proteins is a significant limiting factor in our ability to validate all candidates. To address this limitation, we focused on proteins with available antibodies. Unfortunately, we were only able to procure 7 antibodies against the endogenous proteins highlighted by our analysis. All of them were tested in our experimental setting doing western blot analysis in adult flies. We presented results with the detection of 4 proteins (Me31B, eIF4E, Rump and Syncrip). To answer this comment of Reviewer#1, we have now explained this in the revised manuscript by adding the following sentence on (p.11, line 271): "Since these interactions were validated in vivo, we were unfortunately restricted to candidates for which antibodies were available for the endogenous protein.". We acknowledge that including more candidates from the list, especially those later followed up in functional studies, would add value to the figure, and we hope to pursue this in future work as more resources become available. Unfortunately, specific antibodies for some of the highlighted candidates, such as eIF4G1 and Rin, are not currently available for Drosophila melanogaster. To address this limitation, we conducted co-transfection experiments using tagged versions of Dicer-2 along with tagged versions of eIF4G1, Rin, and Me31B. Me31B served as a positive control, as its interaction with GFP::Dicer-2^{WT} has already been validated in vivo. Tagged Rin, eIF4G1 and Me31B co-immunoprecipitated with Dicer-2 in S2 cells. We included two Rin mutants affecting the phosphorylation at the serine 142 (non phosphorylatable and phospho-mimic) as it has been shown that its localization to stress granules is phospho-dependent. Data are presented in Figure 4B-C and in the Results section p. 12, line 291.*

The co-IP of the large isoform of Syp with Dicer-2 is not reproduced in the experiment shown in Figure 3B, arguing against a very stable interaction.

Reply: *We acknowledge that in the former Supplementary Figure 3B, the large isoform of Syp was indeed not co-immunoprecipitated with Dicer-2. However, as shown on former Figure 3C (new Figure 4E), the interaction of Syp with Dicer-2 seems to be RNA-dependent. Therefore, as an indirect interaction, it would indeed make sense that this interaction would be less stable than a direct protein-*

protein interaction (such as that of Dicer-2/R2D2 for instance). The western blot results shown in this manuscript are representative results of several experiments, and in the new Suppl. Fig. 4A, which corresponds to the uncropped version of former Suppl. Fig. 3B, the larger isoform of Syp is actually found in the elution for The RNase III mutant version of Dicer-2. However to address this comment we have now included the observation that the Syp coIP was not always reproducible in the revised manuscript (p. 12, line 279).

Of note, the presence of a small protein interacting with Dicer-2 was detected using anti-Syncrip after RNas A treatment. However, it has just been brought to our attention that no small isoforms of Syncrip have previously been described at this size, and it does not correspond to any of the 14 predicted isoforms of Syncrip. Consequently, we retained the range of sizes described for Syncrip in the western blot shown in Figure 4. In Supplementary Figure S4, the western blot showing small molecular weight has been kept but annotated as we cannot discriminate if it is non-specific, a cleavage or degradation product. The text has been updated accordingly to reflect this explanation.

The data on RNase-treated samples is described only very superficially. How many of the earlier hits are reproducible? which ones? Also what is the identify of RNase dependent interactors vs RNase independent interactors. The authors should include a proper description of the data. Just showing a Venn diagram does not provide a lot of information.

Reply: *We acknowledge that the data on the MS analysis after RNase A treatment could have been presented in greater details. As we had obtained co-IP results which, in our opinion, provide stronger evidence of RNA-dependent or RNA-independent interactions for some of the candidates, we opted to present this dataset in a more concise manner. Expending this analysis in the same manner as the new Figures 1-2, though certainly interesting, would have considerably lengthened the manuscript and risked becoming repetitive, which is why these data were included as supplementary materials.*

That said, we do agree that a Venn diagram alone does not provide a lot of information, which is why we have represented these data in the revised manuscript as annotated Volcanoplots instead (see new Suppl. Fig. S4D-E). In addition, we have modified the text of the revised manuscript to include the identity of some candidates that were identified again either with and/or without RNase A treatment in this second MS analysis, such as PNPase, Veneno, Homer, FASN2 and Lost. To strike a balance between keeping the manuscript concise and adding relevant information, both the raw data of this MS analysis and the results of the statistical analysis have provided as supplementary figures. This allows interested readers to explore the dataset further while keeping the main text focused. We will make all raw data and scripts available on Figshare with a DOI that will be made available upon publication of the manuscript (temporary private link: https://figshare.com/s/442b2f133f6c16b538cb).

The last sentence of the abstract is an overstatement. The authors have not formally proven that the changed virus levels upon knockdown of selected candidate genes are mediated through the interaction

of that protein with Dicer-2 in RNAi (or beyond). Currently, the protein-protein interaction and the antiviral phenotype are two independent observations. Would the antiviral or proviral phenotype of candidates still be visible in Dicer null mutants or can the authors show that the knockdown affects Dicer-2 function (such as siRNA binding, dsRNA cleavage). The authors should show using such genetics or biochemical assays the role of the interactor in Dicer-2 antiviral activity.

Reply: *We appreciate the Reviewer's constructive comments regarding the connection between protein-protein interactions and the antiviral phenotype, as well as their suggestions for additional experiments to clarify the role of candidate interactors in Dicer-2 antiviral activity. In response, we have replaced this sentence by "This work highlights protein complexes assembled around Dicer-2 in vivo, and provides a resource to investigate their contribution to antiviral RNAi and related pathways.".*

To address the concerns of Reviewer#1 regarding the lack of genetics/biochemical assays to show the role of the candidates in relation to Dicer-2, we conducted several additional experiments aimed at gaining further insight into the underlying mechanisms. Some of these results are presented in Supplementary Figure S5, showing the impact of partner knockdowns on viral protein levels. Notably, we observed increased levels of DCV RdRP protein in eIF4G1 knockdown cells, which correlated with an increase of viral load. Since eIF4G1 is a key component of cap-dependent translation, it is possible that in these conditions, cap-independent translation (e.g., IRES-mediated) is favored which is the translation process for DCV (PMID: 25416947). However, in this experimental setting, we did not observe a decrease in global protein levels, as indicated by Ponceau staining of all membranes used for western blot analysis (image below).

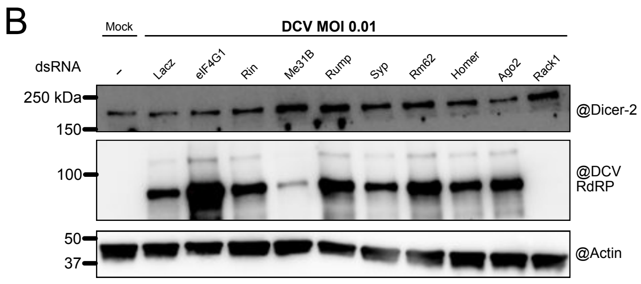

Supplementary Fig S5B

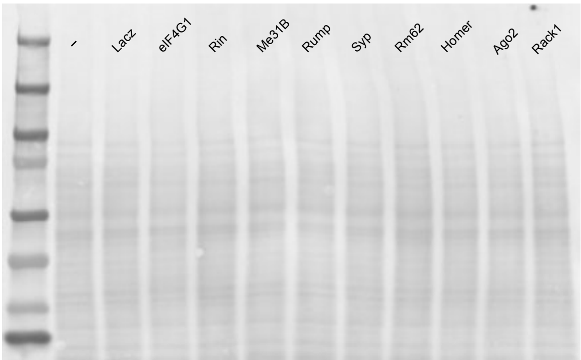

Ponceau staining for membrane S5A Fig.

- *Testing the endo-siRNA pathway:*

We evaluated the impact of our candidate genes on the endo-siRNA pathway using specific sensors for endo-siRNAs (endo-si1 and endo-si2). None of the candidates, except AGO2 (as expected), affected this pathway, suggesting that their antiviral phenotype is unlikely to involve the endo-siRNA pathway. Results are presented in Supplementary Fig. S4A to show that KD of AGO2 impacts the endosiRNA pathway and cited in the main Result section p.15, line 358.

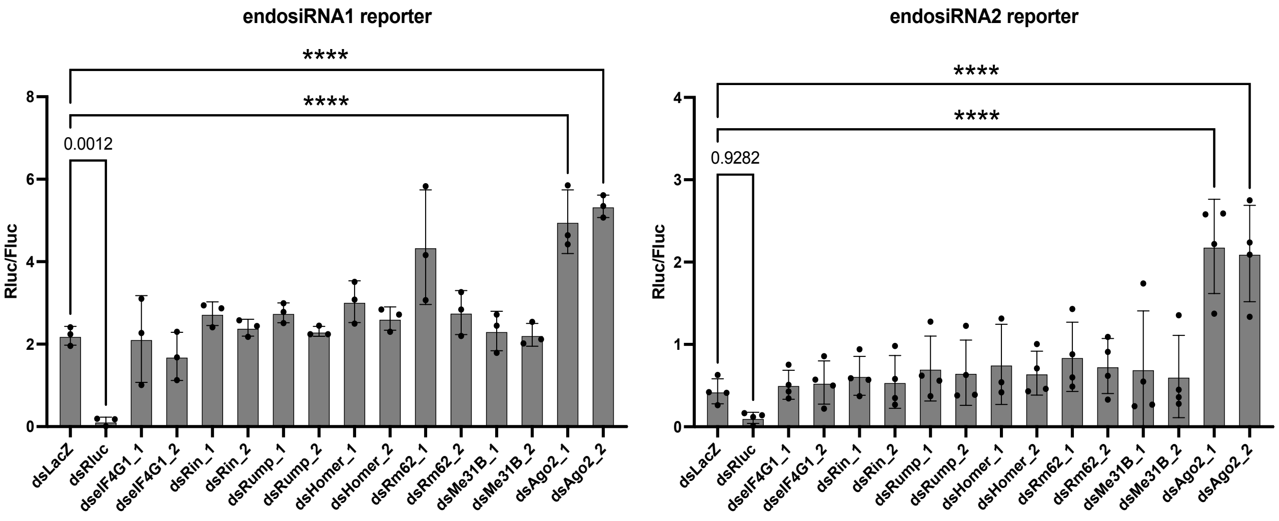

Figure to the reviewer comments: *S2 cells treated with dsRNA were transfected with expression plasmids encoding firefly and Renilla luciferase. Renilla luciferase constructs contain sequences targeted by endosiRNA1 or 2 in the 3'UTR. Renilla luciferase counts were normalized using firefly luciferase counts. Mean ± Standard deviation (SD). Statistical significance was analyzed using one-way ANOVA followed by Dunnett's multiple comparison test. "****" for p < 0.0001.*

- *Northern blot detection of endo-siRNA production:*

Furthermore, we attempted to detect endo-siRNA production via northern blot. However, this experiment proved technically challenging, and we were unable to observe a clear difference (results below). The detection signal is extremely weak, making it unreliable and difficult to quantify accurately. We believe that the endo-siRNA sensors provide a more reliable assessment of this pathway, which is why we only added those in the revised manuscript.

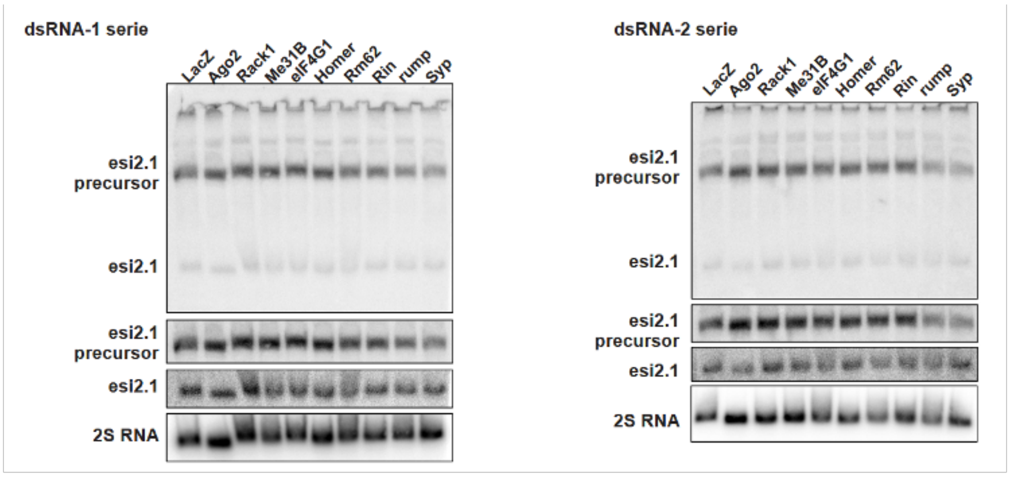

N = 1

25 µg total RNA
Samples extracted 8.10.2024 - Jenny

- *DCV sensor assays for vsiRNA activity:*

To assess the impact of candidate knockdowns on viral siRNA-mediated inhibition of DCV replication, we attempted to produce two DCV-specific sensors composed actin>Renilla-Luciferase with two

different regions of the DCV virus at the 3'UTR. S2 cells were co-transfected with actin>firefly-Luciferase as control and to normalize expression. However, during DCV infection, we did not observe inhibition of the sensors as illustrated below. Treatment with dsRNA targeting Renilla-Luciferase inhibits the expression of the sensor as expected and served as positive control. However, an unexpected upregulation of the sensor was observed in dsLacZ treated cells infected by DCV versus mock. We suspect that DCV1A, a viral suppressor of RNAi encoded by DCV, interferes with the efficiency of vsiRNA-mediated inhibition in our conditions. As this experiment seems to require some optimization, we unfortunately were not able to include it in the manuscript.

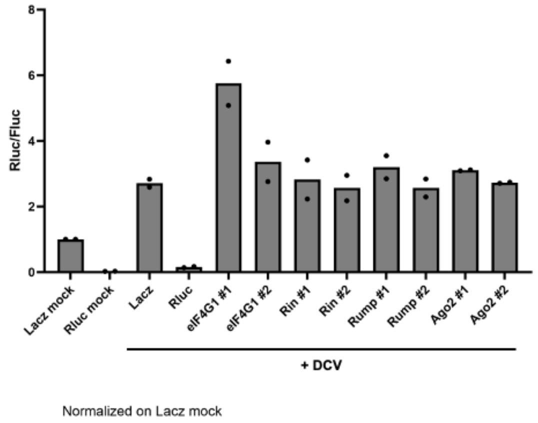

Figure to the Reviewer comments: *DCV Renilla-Luciferase sensor targeting DCV region 4705-5185 (RdRP region). S2 were treated with dsRNA (6 µg/well) - 24 wells with 5.10^5 cells/well - for 3 days. Cells were infected at day3 with DCV MOI 0.1 and transfected at day4 with Luciferase constructs. The Luciferase assay was done at day5. Adapted from PMID: 17079687*

- *DCV vsiRNA detection via northern blot:*

We planned to conduct northern blot analysis for DCV-derived siRNAs. However, to date, no published studies have demonstrated DCV siRNA detection via northern blot.

We finally had to recognize the limitations of using knockdowns in S2 cells, as target proteins are partially reduced rather than completely abolished (knockdown approaches with dsRNA treated cells). This makes investigating small RNA detection by northern blot in S2 cells particularly challenging.

We hope that these additional analyses and insights address the Reviewer's concerns. While the mechanistic link between the antiviral complexes and Dicer-2 interactions requires further investigation, these experiments have provided valuable context and clarified some potential pathways. We believe we can exclude an impact on the endosiRNA pathway of the candidates tested except for AGO2. We are committed to pursuing future studies that leverage alternative approaches, such as mutant fly models and high-throughput sequencing (small RNA-seq), to further elucidate the role of these interactors in Dicer-2 antiviral activity. However, we think that, as the present research paper is intended

to be a valuable resource rather than a detailed mechanistic investigation of these candidates, additional experiments would fall beyond the scope of this paper.

Reviewer #2: (No Response for Part II - Major issues)

General reply: *We thank Reviewer#2 for his/her positive evaluation of our study's design, controls, and execution, as well as for recognizing the robustness of our datasets. We appreciate the Reviewer's suggestion to provide further mechanistic insights into how the identified interactors function in viral infection or interact with Dicer-2. We have addressed these points in the revised manuscript, incorporating new results for eIF4G1 and Rin. Additionally, revisions made in response to the major issues raised by Reviewer#1 aim to clarify the manuscript further. We hope these additions enhance the utility of our study and meet the Reviewer's request for deeper insights.*

Reviewer #3: (No Response for Part II - Major issues)

General reply: *We thank Reviewer#3 for recognizing the comprehensive design and execution of our study. We acknowledge the comment regarding the lack of mechanistic follow-up on the role of interactants in Dicer-2 function. While our primary objective was to provide a robust, hypothesis-generating dataset, we have addressed concerns raised by Reviewer#1 by improving the analysis. Specifically, we have separated the analyses for non-infected and DCV-infected conditions.*

Part III – Minor Issues: Editorial and Data Presentation Modifications

Reviewer #1:

It is unclear throughout the manuscript which negative control line has been used to compare the interactome to? The WT or GFP control or an average of the two. The authors should specify this.

Reply: *To eliminate any potential confusion between CantonS (CS) and wild-type (WT), we have updated the control name throughout the text and figures to "Ctrl-WT." For the control lines, since all datasets are compared to the CantonS background and ubiquitous GFP expression, we have labeled them as "Ctrl-WT + Ctrl-GFP," with corresponding updates made in the main text and figures.*

The authors should specify early in the result section that this MS analysis has been performed in triplicate, which strengthens the case of statistical robustness of data.

Reply: *We have followed the suggestion from Reviewer#1 to mention that the mass spectrometry experiment has been performed in triplicate. This is now specified on p.6, line 149 of the Results section, as well as in the Material and Method section "Viral infection and immunoprecipitations for MS analysis".*

Why are some genes depicted as individual isoforms (such as Homer & Homer RB) whereas other gene isoforms are collapsed into a single entry (such as syp). As this may skew data analysis, it is recommended to have a homogenous approach across all genes.

Reply: *This was unfortunately due to the fact that some spectral counts for different protein isoforms were associated with the same gene name (e.g. Syp) and some with specific names for the different isoforms (e.g. Homer vs Homer-RB) in our databases, which meant that we apparently did not remove all isoforms as intended. We have corrected this by adding a step before the production of all Venn diagrams and heatmaps to remove isoforms homogeneously. The problem should now be solved.*

The rescue phenotypes of the wild type and mutants (shown in Fig S1B) should be explicitly stated rather than referring to the literature (line 133-134)

Reply: *We have taken this comment into account and this is now explicitly stated in the Results section p.7, line 155 in the following sentence: "The impact of the mutations on RNAi efficiency can be observed in Supplementary Figure S1B, as the flies contain the w^{IR} transgene that alters their eye color from red (indicating an impaired RNAi pathway) to white when RNAi is working properly."*

The term "early" steps of dsRNA recognition (e.g. line 131 and 198) is misleading as it implies a temporal component of the experiment. Yet, the timeline of the experiment has been identical for all constructs.

The idea that the mutant are stuck in processing may not necessarily reflect time and should be rephrased.

Reply: *We acknowledge that the term "early" might be confusing, and have rephrased it in the corresponding sentence (Results section p.7, line 151) to avoid implying a temporal component of the experiment. Instead, we explain that the mutants should "provide an overview of the interactome of Dicer-2 while its activity is reduced but its interaction with dsRNA is increased, as both mutations should increase the proportion of Dicer-2 proteins bound to it, due to slower or impaired processing". In addition, we noticed that the term "early steps" was also mentioned in the introduction, and therefore we replaced it by "To stabilize interactions between Dicer-2 and the dsRNA, and thus potentially identify interactants that play a role in the viral dsRNA sensing by Dicer-2, we also used two Dicer-2 mutants able to sense dsRNA but unable to process it." (Introduction section p.6, line 124). This revised phrasing emphasizes the successive stages of dsRNA recognition without suggesting a specific timeline, aligning with the experimental design where the timeline was identical for all constructs.*

Fig 2B and 2C. What does "fold change" refer to? Spectral count? Relative protein abundance. Please specify

Reply: *The above-mentioned figure has now been changed in the revised manuscript, but we have taken this comment into account in all new heatmaps, which represent the number of spectras identified in the MS analysis. This has been indicated in the corresponding figure legends.*

Fig 4B: doublecheck the Y-axis. Currently the data suggests a n up to 10^10 increase in viral RNA levels, which I can barely believe to be true. Also, is the data normalized against LacZ condition? In that case, please correct in the methods, that you plot the delta delta CT values.
Fig 5C: I assume the data is shown on a log scale which is not indicated in the y-axis description.

Reply: *We would like to answer the comments related to Figure 4B and 5C together, as both figures represent the results obtained from a screen. The DCV/RP49 (RP49 is a housekeeping gene) ratio has been plotted (ΔCt), after correction using a linear mixed model to account for variability due to plate, line, row, or replicate effect, which can be non-negligible in these types of screens. Although we initially wrote in the Y-axis legend that this was the "normalized" ratio, we were referring to this ratio being corrected using our linear mixed model, however this data was not normalized against LacZ and is therefore not a ΔΔCt but indeed just the ΔCt. We understand that this might have been unclear and have taken steps to make this clearer by (1) switching the word "normalized" by the word "corrected" and (2) mentioning that we used a linear mixed model to normalize the data in the figure legend. We have also homogenized the Y-axis legends, which was represented in absolute number in the first figure but in log scale for the second, which made it unclear. The axis is now represented in log scale for both figures. Finally, we have added more detail for this normalization in the Methods section of the revised manuscript.*

In the interest of transparency, we would like to disclose that while looking into the R scripts used to plot the data to answer this comment, we noticed that there was an error in our former Figure 5C. Indeed, we had plotted the predicted model resulting from our normalization, which represent the trend that the data would follow if all effect not explained by the model was removed. However, our intention was to plot the observed data after normalization with the model, not the model itself. We have therefore corrected this mistake in the new Figure 6C, where the data is now plotted correctly. This did not affect the results, as the general trend of the model was very similar to the actual normalized data. We apologize for this mistake. We will make all raw data and scripts available on Figshare with a DOI that will be made available upon publication of the manuscript (temporary private link: https://figshare.com/s/442b2f133f6c16b538cb).

FigS5: I am surprised about the apparent randomness of GO terms that appear in the wild type versus mutant analysis. Does that mean that the data is rather noisy and is the overall GO analysis shown in Fig1 then not heavily biased by the Helicase mutant interactome?

Reply: *We agree that this is a valid possibility and have opted to remove the GO term analysis (keep it in Supplementary Table 1). As shown in the revised Figure 2D, GFP::Dicer-2^{Hel1} has significantly more interactants than the other two fly lines following DCV infection. This may result from the Hel1 mutation, which potentially stabilizes interactions with Dicer-2's partners. However, the GO term analysis does not provide additional insights into the Dicer-2 interactome(s), and its inclusion is therefore not justified.*

Line 268: Where does the number 448 come from, the figure shows 317 proteins

Reply: *This comment refers to a paragraph from the previous MS analysis. As this data was re-analyzed in the revised manuscript, these values are no longer present in our revised analysis.*

The authors should discuss the limitation that their assay profiles interactomes in a blend of different tissues from males and females and that interactomes may differ in distinct organs or cell types which cannot be captured with the assay employed in this study.

Reply: *We acknowledge the limitation that our assay profiles interactomes in full flies, using both males and females (20 males + 20 females in the case of MS experiments), which may hide tissue-specific interactions. This approach does not capture potential differential interaction profiles in distinct tissues or cell types. We have modified our revised manuscript to include this in the Discussion section, see p. 19 line 465: "Furthermore, while our analysis highlights the connection between Dicer-2 and oogenesis, which we were able to detect even though it was based on a mixed population of male and female tissues, it might not capture other tissue-specific interactions. Investigating specific tissues, such as the fat body or gut, which are targets of viral infection, could potentially uncover differential interactions and functional roles of Dicer-2."*

I have some suggestions for textual adaptations: I recommend to carefully spell-check the manuscript or let a native speaker proof-read the text.

- Line 126: delete "the dynamics of" the study compares two steady states (infected and non infected) not a dynamic change of interactomes in time or space
- Line 170: help us to better understand
- Line 188 delete "s" in RNAs degradation
- Line 197: replace "be" with "are"
- Line 206: remove "of them"
- Line 213: replace this data with these data
- Line 512: Sentence is not finished
- Line 525 replace "on RNAi" with "an RNAi"

Reply: *We thank Reviewer#1 for pointing out these typos to us. We have corrected the manuscript accordingly.*

Reviewer #2:

1. The included confirmation of interactions by western blot is an important validation, but would be more convincing if the authors indicated in the legends how reproducible the result was, that is, how many times was each western performed?

Reply: *We apologize for this oversight, the representative number of independent experiments has now been specified in the figure legends.*

2. Given that this paper is focused on Drosophila, in addition to citing the mammalian paper on Arc1, it would be appropriate to cite the sister paper about Drosophila Arc1(Ashley J, Cordy B, Lucia D, Fradkin LG, Budnik V, Thomson T. Retrovirus-like Gag Protein Arc1 Binds RNA and Traffics across Synaptic Boutons. Cell. 2018;172(1-2):262-74 e11. doi: 10.1016/j.cell.2017.12.022.)

Reply: *We thank Reviewer#2 for bringing this paper to our attention, we have now cited this paper on p. 8, line 185.*

Reviewer #3:

Suggestions and comments:

- Figure 1B: please define CS

Reply: *In response to this comment, we have changed the "CS" abbreviation in the corresponding figure to the full name of this fly line ("CantonS"), which is defined in the text. As CantonS is used as the wild-type line, we replaced "CS" by "WT" in all figures.*

- Figure 1C: The outer circles indicating significance are not clearly visible

Reply: *In response to this comment, we have changed the color scale of Average number of spectra (AvgSpec) from black-yellow to yellow-blue, to be more easily distinguished from the BFDR color scale, which is black-blue. We now believe that it is easier to distinguish the outer circles indicating significant.*

- Figure 1E: Please define the line widths and colors

Reply: *In response to this comment, we have defined this in the legend of the corresponding figure.*

- Figure 2D: why are there two dots for Dicer-2?

Reply: *To answer this question from Reviewer#3, one of the two dots initially represented the spectras matching to Dicer-2 only and the other to the spectras matching either Dicer-2 or GFP, which were assumed to come from GFP::Dicer-2. As we agree with Reviewer#3 that this might be confusing for the reader, we have removed the "Dicer-2 + GFP" dot in the revised manuscript.*

- Figure 3: staining for a housekeeping protein would be useful.

Reply: *We recognize the importance of loading controls for WB experiments. However, in the context of a co-immunoprecipitation experiment, loading controls are often considered optional, especially given that these experiments already involve multiple antibodies staining for the same samples. In our opinion, using the input of the IP samples as a control is more informative than staining for a housekeeping gene.*

Indeed, as the input is a fraction of the total lysate, taken before the addition of beads, antibodies, or other reagents, this sample serves as a quality control, ensuring that the starting material is consistent across different samples or experiments. While a housekeeping gene might be present in similar quantities across samples, the targeted protein could still vary significantly between samples. If the target protein is homogeneous across lysates, and the same quantity of eluate has been loaded on the gel in all lanes, the addition of a staining for a housekeeping gene may be redundant.

Finally, staining for a housekeeping gene could be considered a negative control, as it would not be present in the eluate after co-immunoprecipitation with Dicer-2. Even so, we have always performed the immunoprecipitation with either a Ctrl-GFP or Ctrl-WT. Negative controls have therefore already been included in these experiments.

- Figure 4: Knockdown of the positive control AGO2 does not affect viral replication, which the authors attribute to using RNAi to knock down an RNAi factor. Yet, such RNAi-of-RNAi approaches have been successfully used before, also in relation to viral infection. Also, the entire screen is to some aspect biased in the same way, RNAi is used to knock down genes

that are likely involved in RNAi. The possibility that this would lead to false negatives merits discussion.

Reply: *RNAi-based knockdown of RNAi factors, such as AGO2, may introduce biases and potential false negatives, as noted in the text (p.15, line 358). While this approach has been used successfully in some studies, our experimental settings suggest limitations due to the experimental conditions, including viral load and time points. This justify that we used two approaches to test selected candidates in drosophila S2 cells and in vivo.*

- Figure 4: please define the box plot (mean, whiskers, etc) in the legend.

Reply: *In response to this comment, we have defined this in the legend of the corresponding figure.*

- All figures: please define the statistical test in the legends.

Reply: *In response to this comment, we have defined this in the legend of the corresponding figure.*

- The prefix sh and ds is not consistently used (shAGO2 and shmCherry)

Reply: *We have used both dsRNAs and shRNAs targeting AGO2: dsAGO2 in the case of the screen in S2 cells and shAGO2 in the case of the in vivo screen. The sh transgenic lines used are TRiP lines containing a short hairpin RNAs (shRNAs) of 21-bp targeting sequence embedded into a micro-RNA (miR-1) backbone (Ni et al. 2011, PMID: 21460824), while the dsRNAs against AGO2 used in S2 cells are in vitro* synthesized double-stranded RNA molecules. *To take into account this comment, we made sure that dsAGO2 was consistently used in the case of S2 cells and that shAGO2 has also been consistently used for the in vivo screen. We hope that this clarifies the matter.*

- Survival curves: please report the number of flies and number of replicate experiments.

Reply: *We apologize for this oversight. The number of flies is now indicated on the survival curves (Supplementary Figure S6). We have also clarified that the survival analysis is based on 3 to 5 independent experiments conducted in three separate batches (legend of Figure 6). These batches were necessary due to the number of crosses and the flies required for injection on the same day, including all control lines. For each independent experiment and condition (TRIS versus DCV), 20 flies from each drosophila line were used (5 males and 5 females per tube, in duplicate). Information have been added in the Materiel and Methods section.*

- I was surprised to find the strong significant effects in the survival curves, given the that some of them are, in fact, overlapping (e.g., Rm62, Rump, Rin). Please doublecheck the statistics.

Reply: *We have double-checked the statistical analysis and confirm that the results presented are correct. Each experimental batch was analyzed using the Log-rank test, and the Benjamini-Hochberg (BH) correction was applied for multiple comparisons of survival curves within each batch. For transparency, we will make all raw data and the scripts used for analysis available. We will make all raw data and scripts available on Figshare with a DOI that will be made available upon publication of the manuscript (temporary private link: https://figshare.com/s/442b2f133f6c16b538cb).*
