# Supplementary material for: In vivo Dicer-2 interactome during viral infection reveals novel pro and antiviral factors in Drosophila melanogaster
Source: PLoS Pathog. 2025 May 7;21(5):e1013093. doi: 10.1371/journal.ppat.1013093 (PMC12058146; doi:10.1371/journal.ppat.1013093)
Supplement: Attachment — Submitted filename: 2-2025_reviewComments.pdf [file ppat.1013093.s015.pdf]

Manuscript ID: PPATHOGENS-D-24-00621R1

In vivo* Dicer-2 interactome during viral infection reveals novel pro and antiviral factors in *Drosophila melanogaster

By Rousseau et al.

We appreciate the constructive feedback and are pleased that all three reviewers acknowledged the substantial improvement of our manuscript. Below, we provide a detailed point-by-point response to the reviewers' comments and outline the revisions made to address them. This revision includes data incorporation in figures, textual modification and extended discussion, and we believe that we have fully addressed the remaining concerns. We look forward to the review process and remain available for any further clarifications.

Point-by point response to the Reviewers' comments: All modifications in the manuscript are highlighted in red

Part I - Summary

Reviewer #1: I would like to compliment the authors for this thorough revision. Readibility and coherence has improved significantly. I appreciate the effort that has been put to address many of the reviers' concerns. I only have a few minor points of feedback.

General reply: *We would first like to thank Reviewer#1 for their constructive comments. We are especially grateful for the valuable suggestions on how to improve both the manuscript and our analytical approach. The feedback provided helped us refine our data analysis strategy, enhance the clarity of our findings, and ensure that our conclusions are well supported. Below, we address the minor points in detail.*

Reviewer #2: In this revised manuscript the authors have responded to prior criticisms primarily by changing the way they analyzed their mass spectrometry data, and including additional transgenic flies expressing GFP alone, to evaluate non-specific interactions. While nicely increasing confidence in the identified interacting proteins, for the most part the factors identified remain the same.

As in the prior submission, the authors include experiments to validate the observed interactions, using immunoprecipitation experiments and western analyses, and they also perform knockdown experiments to evaluate effects on viral load and survival. The latter experiments set the stage for further studies to understand the mechanism by which these factors affect antiviral defense, albeit such studies are not included in this publication.

Regardless, it is still my opinion that the robust datasets will serve as a great resource for future studies by researchers studying Dicer and the antiviral response, as well as those with a general interest in RNA processing.

General reply: *We thank Reviewer#2 for their valuable input and for recognizing the rigor and robustness of our study. We appreciate your acknowledgment of our refined data analysis. While this study does not provide mechanistic insights, we agree that it serves as a robust dataset for future research. We have expanded the Discussion to contextualize the potential*

roles of these interactors in Dicer-2-independent RNAi function and other antiviral pathways, especially the cGLR/STING pathway.

Reviewer #3: In this revised manuscript, the authors analyzed the Dicer interactome in adult flies using mass spectrometry, in non-infected and virus infected flies. As already indicated in my original review, the work is well designed and well executed, and the data are comprehensively reported. The paper provides interesting leads to follow-up studies of Dicer-2 interactors.

In my review of the original submission, I expressed doubts about the appropriateness for PLOS Pathogens given the lack of mechanistic insight. My assessment still applies; interesting questions raised in the introduction are not addressed in the manuscript. However, as the authors were given the opportunity to revise their manuscript and since they have extensively revised it and addressed my comments, I now support publication in PLOS Pathogens. There is, however, one essential experiment that needs to be done.

General reply: *We sincerely thank Reviewer#3 for their thoughtful feedback and for recognizing the improvements made to the manuscript. We note the concern of Reviewer#3 regarding the lack of direct assays monitoring small RNA production. While we agree that small RNA-sequencing is the most direct approach to assess Dicer-2 activity, we believe that this experiment is unnecessary given our existing data. To address this concern, we conducted an endo-siRNA reporter assays which demonstrates that this pathway remains unaffected by the candidate genes tested, thereby refining their potential roles. Below, we explain the changes made in the Part II - Major Issues.*

Part II – Major Issues: Key Experiments Required for Acceptance

Reviewer #1: none

Reviewer #2: (No Response)

Reviewer #3: The effect of the interactors on Dicer-2 enzymatic activity has not analyzed. An analyses of siRNA profiles (viral and endogenous) upon knockdown of key interactors would greatly improve the value of the manuscript as it is the most direct readout of Dicer-2 function.

We understand the rationale behind this request. However, based on our existing data, we believe that small RNA-sequencing would be outside the scope of this paper. Indeed, our results already show that the main candidates do not impact endo-siRNA1 and endo-siRNA2 activity. While we could focus on viral siRNAs, the RNAi-independent functions of Dicer-2, combined with the experimental cost limiting the number of candidates we can test and the challenges of using RNAi to knockdown a protein involved in RNAi, make the likelihood of uninformative results too high.

To address the previous comments related to the link between Dicer-2 and the candidates during the first round of reviewing, we had previously performed additional northern blot analysis of siRNA production and reporter assays to assess siRNA-dependent silencing. The endo-siRNA reporter assay provides direct evidence that RNAi functionality remains unaffected. Given that none of the candidates show a measurable effect on endogenous siRNA-mediated silencing, it remains unclear which interactor would be the best choice for small RNA sequencing.

However, in an effort to better integrate the role of the candidates in relation with Dicer-2 into the manuscript, we have made the following changes:

- *We added the endo-siRNA2 reporter assay (previously in Fig. S5A) as an additional panel in Figure 5 to highlight that the interactors tested do not affect endogenous siRNA-dependent silencing. We performed a total of 5 independent experiments using the endo-siRNA2 reporter to confirm this.*
- *We incorporated these results, depicted in Figure 5B, at the end of the Chapter "Identification of new proviral and antiviral factors in drosophila S2 cells" - Results section, page15 line 381.*
- *The previous schematic representation Figure 5A describing the knockdown screen was removed as it is a standard procedure. Instead, we add a schematic panel in Figure 5B to illustrate the endo-siRNA sensor.*
- *References to Figure 5 in the text have been corrected according to the changes.*
- *In the Discussion section, we added a paragraph discussing our findings and alternative functions of Dicer-2 beyond siRNA production (page 18, line 453). We propose that some of the interactors may contribute to cGAS-like receptor (cGLR)-mediated viral sensing, referencing studies (PMID: 21889502, 29317541, 18953338, 39495785, 34965418, 34261128, 38377349) (page 20, line 514).*

Part III – Minor Issues: Editorial and Data Presentation Modifications

Reviewer #1: I find the MDS analysis in response to one of my earlier questions a rather useful piece of data to obtain a first impression of overall trends in the data. I would highly recommend to include this panel in supplemental figure 1, alongside a brief description in the main text.

During the first round of revisions, we have been performed a Multidimensional Scaling (MDS) analysis to visualize the similarity and dissimilarity between mass spectrometry samples in a reduced-dimensional space included in the IPinquiry4 package *(PMID: 3630892). Control lines (CantonS "Ctrl-WT" and GFP-expressing flies "Ctrl-GFP") cluster together but distinctly from the Dicer-2::GFP samples, reflecting their unique characteristics. We have now included the MDS analysis in Supplemental Figure 1C, as suggested, along with a brief description in the main text (Results section, line 167).*

Line 263. Change to …, and the predicted interactions between those candidates was represented… Not all links in a STRING network are truly validated physical protein-protein interactions.

We have replaced, line 270, "interactions" by "predicted interactions".

At various points the authors refer to the S2 cell experiments as "ex vivo" which is a term referring to tissue cultures explanted from living organisms. I propose to change this to "in cells".

We have replaced all instances of "ex vivo" with "in cells" in the text.

The lack of formal experimental evidence if identified interaction partners act on virus replication through RNAi is worth mentioning in the discussion.

We have added a paragraph in the Discussion section to acknowledge the lack of formal evidence that the identified interaction partners act on virus replication through RNAi. Additionally, we have discussed Dicer-2-independent functions beyond RNAi and additional

immune pathways, in particular the cGLR/STING pathway (cf. Part II Major issues, answer to Reviewer#3).

Reviewer #2: (No Response)

Reviewer #3:

Line 149: I think it should suffice to mention numbers of replicates in legends, not in the main text.

The sentence "This experiment has been performed in triplicate." Has been moved to the figure legend of Figure 1B.

Line 180: With reference to Veneno, the authors may also want to discuss the Brosh et al paper (PMID: 35858337), showing an antiviral phenotype of a truncated Veneno allele.

We appreciate the suggestion to consider the work of Brosh et al. (2022), which identified a novel antiviral function for a truncated allele of Veneno in Drosophila melanogaster against Drosophila A virus (DAV). This study highlights an intriguing mechanism where gain-of-function mutations through TE insertions can lead to novel antiviral factors, independent of canonical RNAi pathways. We have now referenced these findings in the Results section (line 183). We discuss Dicer-2-independent functions of the RNAi in the Discussion section.

Line 199: a case is made that Lost is especially enriched in Helicase mutant. However, this conclusion is based on the p value, but the difference in Lost enrichment seems to be minor based on the fold enrichment (which is more informative than the p value). I suggest rephrasing.

We wrote the above-mentioned sentence because we noticed that the ranking of Lost in our analysis had changed drastically. However, we acknowledge that this is mostly due to the difference in p-value, which is less informative than the fold-change. We have therefore rephrased this sentence to reflect this in the Results section (line 202).

Line 227-228: "these interactants include the viral RNA-dependent RNA polymerase (RdRp) from DCV (ORF1)". I found this phrase a bit ambiguous: the reference to ORF1, which encodes also other viral proteins, suggests that there may be other viral Dicer-2 interactors in addition to the RdRP. Please clarify, or delete the reference to ORF1 in the sentence.

We appreciate the reviewer's feedback regarding the reference to DCV ORF1. We have verified the DCV peptides annotations in the mass spectrometry (MS) data to ensure accuracy. Upon double-checking, we have determined that the peptides detected as "ORF1" matched more specifically the polyprotein known as the DCV Replicase polyprotein, which contains several non-structural proteins that are later cleaved/maturated. In our data, these peptides match almost exclusively the RdRp and DCV1A sequences. This verification ensures that our conclusions remain precise and correctly reflect the data. To avoid ambiguity, we have removed "ORF1" from the text and have used the Uniprot name "DCV Replicase polyprotein" instead (line 230). We also updated the figures according to the Uniprot annotation (Figures 2, 3 and S3).

Figure 3, please define BFDR

We clarified the term "BFDR" in Figure 3 by adding the sentence "The Bayesian false discovery rate (BFDR) is indicated." (line 1058).

IP mass spec was done on lysates of total animals, but I expect that only a minority of the cells in the entire animal is DCV infected. Hence the majority of cells in the infected samples may in fact be noninfected cells. Could the authors comment on this? How was the inoculum and time of analysis chosen? Did the authors monitor viral loads / number of infected cells at the indicated time point?

Based on our previous studies, we showed that flies injected with DCV 500pfu two days post infection exhibit a high viral load, which reaches a plateau at two days post-infection (PMID: 32843367). In adult flies, DCV infects a wide range of tissues with the fat body (PMID: 18953338), the gut (PMID: 25253354) and most of other adult tissues (PMID: 39688951), especially at two days post-infection. Moreover, the flies start to die only after this time point. We have therefore selected this timepoint for our experiments. The mass spectrometry data reveals that RdRp and DCV1A of DCV are enriched only in Dicer-2 samples with a high number of spectra (top ranked candidate). While none or few spectra are detected in control samples in infected condition, suggesting that GFP::Dicer-2 samples are clearly enriched from infected tissues. We thus think that, although it is true not all cells in the fly will ever be infected, the use of this timepoint was adequate to allow us to draw conclusions on the impact of Dicer-2 mutations on the infection, in comparison with the controls.
